# High-Efficiency Perovskite/Silicon Tandem Solar Cells Based on Wide-Bandgap Perovskite Solar Cells with Unprecedented Fill Factor

**DOI:** 10.1007/s40820-025-01959-y

**Published:** 2026-01-14

**Authors:** Li-Chun Chang, The Duong, Viqar Ahmad, Hualin Zhan, Anh Dinh Bui, Jana-Isabelle Polzin, Armin Richter, Gabriel Bartholazzi, Keqing Huang, Zhongshu Yang, Wei Wang, Yihui Hou, Li Li, Qian Cui, Rabin Basnet, Jianfei Yang, Hong Lin, Guozheng Du, Khoa Nguyen, Dang-Thuan Nguyen, Lachlan E. Black, Daniel MacDonald, Daniel Walter, Klaus J. Weber, Kylie R. Catchpole, Heping Shen

**Affiliations:** 1 School of Engineering, The Australian National University, Canberra, Australian Capital Territory, 2601 Australia; 2 Division Photovoltaics, Fraunhofer Institute for Solar Energy Systems ISE, Heidenhofstrasse 2, 79110 Freiburg, Germany; 3 Research School of Physics, The Australian National University, Canberra, Australian Capital Territory, 2601 Australia; 4 State Key Laboratory of New Ceramics & Fine Processing, School of Materials Science and Engineering, Tsinghua University, Beijing, 100084 People’s Republic of China

**Keywords:** Inverted perovskite solar cells, Self-assembled monolayers, Interface energy-level alignment, Wide-bandgap perovskite, Tandem solar cells

## Abstract

Recent progress in inverted perovskite solar cells (iPSCs) highlights the critical role of interface engineering between the charge transport layer and perovskite. Self-assembled monolayers (SAM) on transparent conductive oxide electrodes serve effectively as hole transport layers, though challenges such as energy mismatches and surface inhomogeneities remain. Here, a blended self-assembled monolayer of (2-(9H-carbazol-9-yl)ethyl)phosphonic acid (2PACz) and (4-(3,6-Dimethyl-9H-carbazol-9-yl)butyl)phosphonic acid (Me-4PACz) is developed, offering improved surface potential uniformity and interfacial energy alignment compared to individual SAMs. Interactions between the SAMs and ionic species are investigated with simulation analysis conducted, revealing the elimination of interfacial energy barriers through precise energy-level tuning. This strategy enables wide-bandgap (1.67 eV) perovskite solar cells with inverted structures with over 24% efficiency, an open-circuit voltage (*V*_oc_) of 1.268 V, and a certified fill factor (FF) of 86.8%, leading to a certified efficiency of 23.42%. The approach also enables high-efficiency semi-transparent devices and a mechanically stacked four-terminal perovskite/silicon tandem solar cell reaching 30.97% efficiency.

**Supplementary Information:**

The online version contains supplementary material available at 10.1007/s40820-025-01959-y.

## Highlights

- By mixing 2PACz and Me-4PACz, an energetically homogeneous buried interface is formed, enabling preferential energy alignment at the hole transport layer/perovskite (1.67 eV) interface, which delivers a certified fill factor of 86.8% and a power conversion efficiency of 23.42%.
- Simulations indicate that at lower interface defect densities (1 × 10^8^–1 × 10^11^ cm^−2^), improvements in FF dominate the device performance, whereas at higher defect densities (1 × 10^12^–1 × 10^13^ cm^−2^), *V*_oc_ is the key factor.

**Supplementary Information:**

The online version contains supplementary material available at 10.1007/s40820-025-01959-y.

## Graphical abstract

**Figure:**
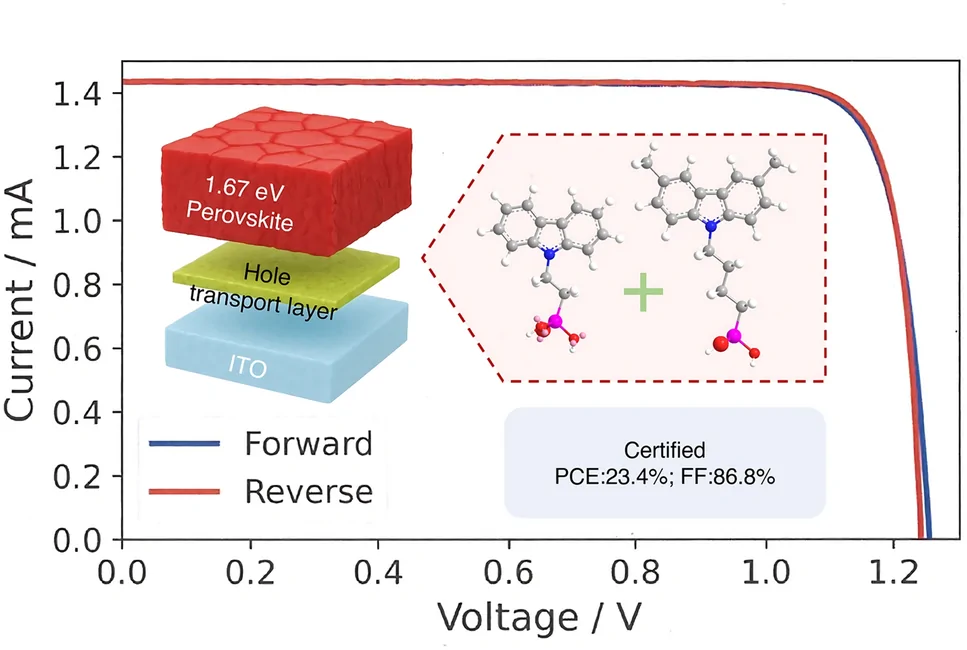

## Introduction

In the past decade, enormous progress has been achieved on the perovskite solar cells, particularly those with inverted structures, reaching a record power conversion efficiency (PCE) of 27.3% [1], closely rivalling the 27.8% record PCE achieved by market-dominant silicon (Si) cells [2]. Si solar cells account for over 97% of annual global production as of the end of 2023 [3]. However, with the theoretical intrinsic PCE limit of ≈ 29.4% [4, 5] and the efficiency growth expected to plateau by 2030 for standalone Si solar cells [6], tandem solar cells combining Si with perovskite cells have emerged as a promising solution to further improve energy yield and reduce levelized cost of electricity [7, 8]. Four-terminal (4-T) perovskite/Si tandem, offer flexibility in bandgap selection and independent cell operation [9], recently achieved record efficiency of 33.10% by combining a perovskite cell with a back-contact (BC) Si solar cell [10]. However, this remains inferior compared to their two-terminal (2-T) counterparts, which have reached 34.6% [11]. Further improving the PCE of wide-bandgap (WBG) (1.65–1.80 eV [12]) iPSCs is essential for enabling efficient perovskite/Si tandem solar cells [13]. Currently, the record PCE of WBG iPSCs (≈1.67 eV) has reached 23.67%, with a *V*_oc_ of 1.249 V, a short-circuit current (*J*_sc_) of 22.44 mA cm^−2^, and a FF of 84.66% [10]. However, WBG iPSCs still exhibit significant *V*_oc_ and FF losses, with deficits of approximately 134 mV and 5.35% absolute (best result), respectively, as compared to the Shockley–Queisser limit *V*_oc_ (1.374 V), and FF (90.85%) [14]. These deficits are larger than those in record efficiency 27.3% iPSCs based on narrow-bandgap perovskite, which show *V*_oc_ and FF deficits of around 62 mV and 4.8%, respectively [1].

The FF is a critical performance metric in solar cells, reflecting the quality of the device's electrical characteristics. The FF is influenced by *V*_oc_, as higher *V*_oc_ often correlates with reduced recombination losses and improved charge extraction, indirectly enhancing FF. The observed deficits are primarily attributed to non-radiative recombination losses at the perovskite interface and phase segregation in the perovskite bulk [15]. Recent advancements in WBG iPSCs focus on defect passivation, such as employing ammonium groups to passivate positively charged vacancies, at the perovskite/C60 interface [16, 17]. However, many passivation layers create a trade-off between *V*_oc_ and FF, due to their non-conductive nature [18]. Consequently, achieving high *V*_oc_ and FF in WBG iPSCs through passivation strategies remains challenging [19]. An effective approach to achieving high *V*_oc_ without compromising FF involves improving the charge transport layer (CTL)/perovskite interface energy alignment [20, 21]. This method mitigates the interfacial recombination by minimizing the energy offset, while maintaining material conductivity [22]. Stolterfoht reported that even a small majority-carrier band offsets (≈ 0.2 eV) at the perovskite/electron transport layer (ETL) interface can lead to enhanced interfacial recombination [22]. For instance, Chen et al*.* found that the dual binding at neighbouring Pb^2+^ defect sites mitigate energy mismatches at the perovskite/ETL interface [23], reducing *V*_oc_ and FF losses caused by band bending [22]. Zhang et al*.* introduced a fullerene-derived interlayer (PCBB-3N-3I), which drives an ordered dipole layer formation to enhance the built-in field. The band bending at the perovskite/ETL interface was enlarged, inducing a vacuum-level downshift (≈ 0.19 eV), thereby lowering the electron collection barrier [24]. Jiang et al*.* demonstrated that surface energetics at the perovskite/ETL interface can be effectively modified using 3-(aminomethyl) pyridine, which induces a built-in electric field in the surface region. This treatment decreased the work function by 0.56 eV and raised the valence-band maximum by 0.71 eV, thereby facilitating electron extraction while simultaneously blocking holes [25]. In contrast, the hole transport layer (HTL)/perovskite interface has been less studied in the *p-i-n* structure, to challenges posed by the perovskite precursor solvent dissolution of the bottom passivation materials [26, 27].

Efficient hole extraction at the bottom transparent conductive oxide (TCO) requires precise HTL energy alignment [28]. SAMs anchor to the TCO through chemisorption with hydroxyl sites [29], creating a dipole that shifts the work function (WF) downward [30], enhancing hole selectivity [31]. While SAMs like 2PACz (WF ≈ 5.0 eV on indium tin oxide (ITO)) have shown promise in narrow-bandgap devices [32], challenges such as non-uniform energy potential and suboptimal alignment persist for WBG perovskites, where the valence-band maximum (VBM) deepens to around 6.00–6.07 eV [43–45]. To address these issues, strategies such as introducing two-dimensional (2D) perovskite layers, interlayers with tailored band alignments, and doping have been explored. For instance, Azmi et al*.* introduced an alkyl amine ligand atop the SAM HTL, which regulated the formation of the 2D perovskite layer, and, in turn, promoted hole transfer by deepening the VBM of the form 2D perovskite at the HTL/perovskite interface [33]. Similarly, Tan et al*.* optimized a *p*-type ITO/perovskite interface through targeted doping of the perovskite bottom surface to enhance interface electronic charge transfer [34]. While interlayer insertion boosts performance, it increases fabrication time and costs, making it less suitable for scalable industrial applications.

Apart from the energy-level mismatch at the HTL/perovskite interface, interfacial energetic inhomogeneities at the hole-collecting front contact also leads to performance losses [35]. One of the primary causes of energy inhomogeneities is the non-uniform SAM coverage, which arises from the aggregation of the SAM on the TCO surface. For instance, density functional theory (DFT) reports lateral 2PACz-2PACz interaction energies (− 0.3 to − 0.1 eV per molecule [31]). This cohesion facilitates capture of mobile 2PACz by dimers/trimers, leading to agglomeration, inhomogeneous SAM coverage, and a non-uniform surface energy/work function distribution [11]. According to the Helmholtz equation, the ΔWF shift of the TCO/SAM stack is directly proportional to the dipole surface density N/A, where the N/A denotes the number of dipoles per area [36]. As a result, the charge extraction efficiency is influenced by the nanoscale and macroscale stoichiometry and distribution of the SAMs, along with their orientation, and packing density. In addition to ensuring a homogeneous surface potential, the HTL must minimize electrical shunts caused by perovskite inhomogeneities, such as pinholes. Despite its significant impact on device performance, regulating the surface potential on the hole-selective side of the perovskite has received little attention. Therefore, a careful design for HTL is needed to enhance surface homogeneity accompanied by a strong bonding with the underlying TCO.

To address the challenges of interface energy alignment and surface potential homogeneity at the HTL/perovskite interface, we developed an efficient HTL based on the mixture of 2PACz and Me-4PACz. We observed that the ratio of 2PACz to Me-4PACz significantly influenced the HTL surface WF and surface potential uniformity in the Kelvin probe force microscopy (KPFM) mapping. By carefully tuning this ratio, we optimized the interface energy alignment to enhance efficiency and achieve a homogeneous potential distribution which aligned with the reduced of the surface roughness as suggested by the atomic force microscopy (AFM) which allows void-less perovskite surface observed in the scanning electron microscopy (SEM). The interactions between cations and anions with SAMs functionalized with additional methyl groups on the benzene ring were probed using Fourier transform infrared spectroscopy (FTIR), steady-state electroluminescence (EL), photoluminescence (PL), and X-ray photoelectron spectroscopy (XPS). Furthermore, we conducted an in-depth investigation into the elimination of undesired energy barriers at the HTL/perovskite interface through precise energy-level tailoring. Replacing 2PACz with this mixture resulted in a pinhole-free perovskite film, and efficient solar cell based on a mixed-cation and mixed-halide perovskite with a bandgap of 1.67 eV, with a high efficiency of 24.07%. The certified PCE reached 23.42%, along with an exceptional FF of 86.8%. The quasi-fermi level splitting (QFLS) suggested negligible loss of carrier transport at the interface. We note that this is the record FF reported for the WBG iPSCs ranging from 1.65 to 1.75 eV (Table S1). This FF represents only a 4.1% deficit compared to the Shockley–Queisser limit, marking the first instance where the FF deficit of the wide-bandgap PSC is smaller than those reported in the low bandgap PSCs [14]. The achievement of a high *V*_oc_ of 1.268 V demonstrates the effectiveness of this approach in simultaneously achieving both high *V*_oc_ and FF, leading to superior device performance. The device modelling results indicate that improper energy alignment at the HTL/perovskite interface creates an unfavourable energy barrier, which hinders efficient charge transfer. By increasing the WF closer to 6.0 eV, the simulated FF showed a significant improvement, particularly at lower HTL/perovskite interface defect densities (1 × 10^8^–1 × 10^10^ cm^−2^), accompanied by an increase in *V*_oc_. The mixed SAM also significantly enhanced the shelf stability of the device, leading to no performance loss of its initial PCE after 1680 h of storage in a nitrogen (N_2_) atmosphere. Moreover, the operational stability of the target device remained at an average of 91% of its initial PCE after 1000 h of continuous 1-sun illumination in a N_2_ atmosphere at 25 °C. Using the champion semi-transparent (ST) perovskite cells fabricated with 2PACz and Me-4PACz mixture HTL, we achieved 30.97%-efficient mechanically stacked, 4-T perovskite/Si tandem configuration. This was accomplished using an industrially prevalent Si bottom cell structure, the tunnel oxide passivated contact (TOPCon) Si cell, marking it the highest efficiency reported for this type of device.

## Experimental Section

### Materials

All materials were used as received without further purification. ITO and fluorine-doped tin oxide (FTO) glasses were purchased from Shang Yang Solar Technology Co., Ltd. Caesium iodide (CsI), caesium bromide (CsBr), lead bromide (PbBr_2_), and lead chloride (PbCl_2_) were purchased from Sigma-Aldrich. Lead iodide (PbI_2_, 99.99%), 2PACz (> 98.0%), and Me-4PACz (> 99.0%) were purchased from Tokyo Chemical Industry (TCI). C60 (Nano-C, 99.9%) was ordered from the company Nano-C. Formamidinium iodide (FAI), formamidinium bromide (FABr), methylammonium iodide (MAI), methylammonium bromide (MABr), methylammonium chloride (MACl), and propane-1,3-diammonium iodide (PDAI_2_) were purchased from the Greatcell Solar Materials. Anhydrous solvents, including N,N-Dimethylformamide (DMF), dimethyl sulfoxide (DMSO), chlorobenzene, methanol, and isopropanol, were purchased from Sigma-Aldrich. Certified refractive index liquid series: AA (n_D_^25°C^ 1.414 ± 0.002) was purchased from Structure Probe, Inc. (SPI) Supplies.

### Fabrication of the Solar Cells

The Si solar cells (2 × 2 cm^2^) were fabricated following the previous reported process [32], fabrication details are included in the Supplementary Information.

Opaque perovskite solar cells with bandgaps of 1.67 and 1.73 eV, as well as semi-transparent devices, were fabricated using previously reported procedures [27, 37], and fabrication details are included in the Supplementary information. The compositions of the perovskite are [(Cs_0.22_FA_0.78_)_0.95_MA_0.05_]Pb[(Br_0.15_I_0.85_)_0.96_Cl_0.04_]_3_ (1.67 eV), Rb-FA_0.75_MA_0.15_Cs_0.1_PbI_2_Br (1.73 eV). The structure of the opaque cell is ITO/SAMs/perovskite/ PDAI_2_/C60/SnO_*x*_/Ag. The structure of the semi-transparent cell is ITO/SAMs/perovskite/ PDAI_2_/C60/SnO_*x*_/indium zinc oxide (IZO)/Ag.

Four-terminal tandem testing was performed by stacking a semi-transparent perovskite top cell over a TOPCon Si bottom cell. A 2 × 2 cm^2^ mask on the Si defined the active area directly beneath the perovskite optical filter. A certified index-matching liquid (series AA, n_D_^25°C^ 1.414 ± 0.002) was applied between the two cells to minimize Fresnel losses, and a textured foil was placed on the perovskite front surface to reduce reflection. The texture foil was manufactured using textured silicone foil featuring an inverted pyramid texture [38]. Each sub-cell was measured independently under simulated AM1.5G illumination.

### Characterization

KPFM mapping, AFM, SEM, steady-state EL, PL, FTIR, grazing incidence X-ray diffraction (GIXRD), XPS, transmittance, QFLS, current–voltage (*I-V*), external quantum efficiency (EQE), and stability measurement details have been included in the supplementary file.

## Results and Discussion

### Tailoring and Homogenizing of Surface Energy Potential

**Fig. 1:**
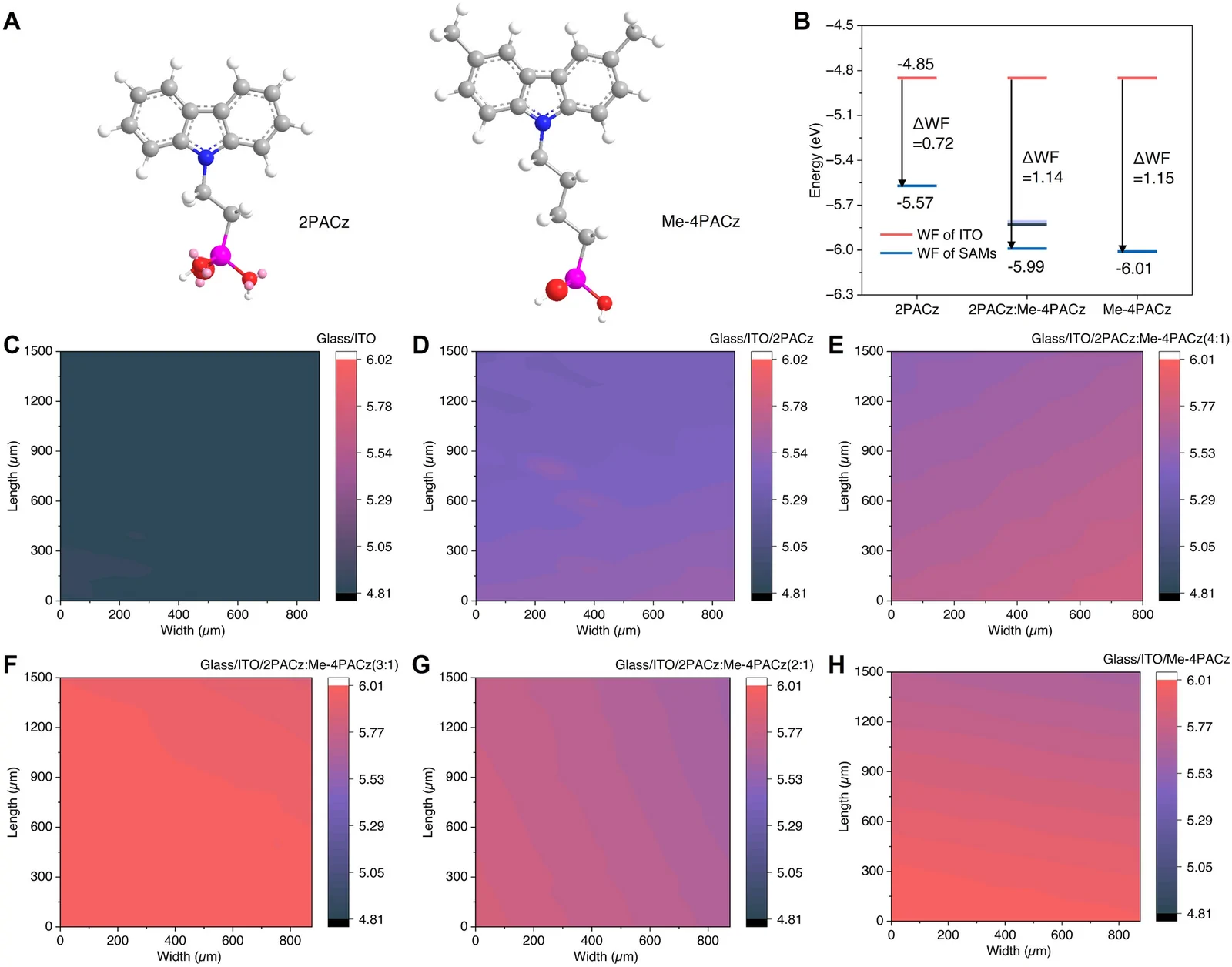
**A** Chemical structure of 2PACz (left) and Me-4PACz (right) (white colour ball: hydrogen atom; grey colour ball: carbon atom; blue colour ball: nitrogen atom; pink colour ball: phosphorus atom; and red colour ball: oxygen atom). **B** Work function diagram of the 2PACz, mixture of 2PACz:Me-4PACz with the ratio of 2:1 (light blue), 3:1 (blue), and 4:1 (dark blue), and Me-4PACz top surfaces on ITO. Work function distribution of **C** ITO, **D** 2PACz, and 2PACz:Me-4PACz mixture with the ratio of **E** 4:1, **F** 3:1, and **G** 2:1, and **H** Me-4PACz

Methoxy and methyl substituents represent common strategies to modify the terminal groups of 2PACz for improved interfacial properties [32, 39]. The methoxy group, with its out-of-plane lone pair, behaves as a Lewis base towards perovskites and has been reported to effectively passivate the buried interface [40, 41]. In contrast, methyl substitution primarily modulates the dipole moment, thereby tuning the energy-level alignment between the HTL and the perovskite absorber, which facilitates hole extraction and transport [41]. Compared with 4-(3,6-dimethoxy-9H-carbazol-9-yl) butyl]phosphonic acid (MeO-4PACz), Me-4PACz exhibits superior material and interfacial properties, including a higher decomposition temperature (by ≈ 146 °C) indicating enhanced thermal stability, a deeper work function (by ≈ 0.26 eV) and valence band (by ≈ 0.11 eV) that reduce the offset with the wide-bandgap perovskite valence band, and a smoother surface morphology with a reduced root mean square (RMS) roughness of ≈ 0.08 nm [42]. From the perspective of interfacial energy alignment, we therefore focused on 2PACz and Me-4PACz as a complementary pair. The molecular structures of 2PACz and Me-4PACz are illustrated in Fig. 1a. Compared to 2PACz, Me-4PACz features an additional methyl (–CH₃) group attached to the benzene ring and a longer methyl chain between the nitrogen atom and the phosphonic acid with a larger WF [43]. We first measured the WF and surface potential homogeneity of TCO layer coated with each SAM and their mixture with different molecular ratios (2PACz:Me-4PACz from 4:1 to 2:1) using Kelvin probe mapping. In this work, commercial ITO-coated glasses were used as substrate. The bare ITO layer exhibits a maximum WF of approximately 4.85 eV (Fig. 1b). When 2PACz is anchored onto the ITO, the maximum WF increases to 5.57 eV, corresponding to a shift of + 0.72 eV. Meanwhile, when Me-4PACz was coated on the ITO, the maximum WF was shifted to 6.01 eV. This is in line with the previous results [43]. As the 2PACz:Me-4PACz ratio changes from 4:1 to 3:1, the maximum WF absolute value from the mapping increases from 5.81 eV (light blue line in Fig. 1b) to 5.99 eV (blue line in Fig. 1b). This increase is likely due to the higher proportion of Me-4PACz, which induces a more significant WF shift on the ITO surface. However, when the ratio decreases to 2:1, the maximum WF value from the mapping decreases to 5.83 eV (dark blue line in Fig. 1b). This reduction is likely due to the aggregation of Me-4PACz as its percentage increases, resulting in unoccupied regions on the ITO surface and subsequently lowering the WF value. The increased WF of the mixture SAMs at the ratio of 3:1 compared to 2PACz and ITO, indicates a stronger built-in electric field [44, 45] and thus potentially enhancing carrier extraction in PSCs [46]. We subsequently characterized the energy levels of the perovskite bottom surface by mechanically peeling off the perovskite film following the reported protocol [47], and measuring with a Kelvin probe. The valence band was determined to be ≈ 5.51 eV (2PACz) and ≈ 6.07 eV (Fig. S1) (Mixture 2PACz:Me-4PACz 3:1), while the work function was ≈ 4.47 eV (control) and ≈ 4.47 eV (target). We observed modest variations in the measured bottom surface energy levels. This is expected because perovskite nucleation on different HTLs and possible carryover of SAM molecules onto the exposed surface that can modify the interfacial dipole [48]. Moreover, the 2PACz work function is larger than the perovskite valence-band maximum (+ 0.06 eV) compared to target (− 0.08 eV), producing an offset (i.e. a hole-extraction barrier), which will be discussed in the simulations section. These values are consistent with reported literature of 1.67 eV perovskite, where the valence band ranges from 5.48 to 6.07 eV, and the work function spans 4.3–5.14 eV [49–51].

To analyse the energy potential homogeneity (Fig. 1c–h), the WF data of each film were further evaluated by calculating the standard deviation (SD) measured area of 1.6 mm^2^ (2 mm × 0.8 mm). The pure ITO film shows the smallest SD (0.01 eV), indicating a uniform energy potential of the ITO layer deposited on the glass. We observed the linear gradient pattern in the mapping images E to H, which can be ascribed to the spin coating in the film preparation which reflect the surface coverage or layer density. The pure 2PACz film-coated ITO substrate exhibits a slightly increased SD, reaching approximately 0.05 eV. This increased SD can be attributed to non-uniform coverage of 2PACz on the ITO layer, resulting from insufficient –OH sites for anchoring, which leaves portions of the ITO surface uncovered. Additionally, variations in the orientation of 2PACz molecules on the surface, influenced by the location of anchoring points and molecular aggregation, further contribute to the inhomogeneity. The Me-4PACz film exhibits an even larger SD of approximately 0.11 eV. These results indicate that energy homogeneity decreases when a single SAM is coated. The higher nonuniformity of Me-4PACz is ascribed to the steric hindrance effect of Me-4PACz due to the increase molecule size to anchor onto the ITO [52]. Despite the higher WF measured at certain points on the Me-4PACz film, the absolute median WF (5.85 eV) is lower than that of the 2PACz:Me-4PACz mixture (3:1) (5.98 eV) due to the exposure of the ITO on the surface and, thus, the non-uniform coverage caused by the aggregation of the Me-4PACz. The mixture with ratios of 4:1 and 2:1 (2PACz:Me-4PACz) exhibits smaller SDs (0.07 and 0.06 eV, respectively) than the pure Me-4PACz film, indicating better uniformity. Notably, when the ratio is 3:1, the film demonstrates uniformity with a very low SD of 0.02 eV, which is very close that of the pure ITO surface. The improved uniformity of the 2PACz:Me-4PACz film at a 3:1 ratio is likely attributed to the size difference (as indicated by the molecule weight and formulas) between the 2PACz (275.24 g mol^−1^ and C_14_H_14_NO_3_P) and Me-4PACz (331.35 g mol^−1^ and C₁₈H₂₂NO₃P) molecules, which allows 2PACz to fit between Me-4PACz molecules, thereby reducing segregation.

To assess how the 2PACz:Me-4PACz blend affects surface morphology, AFM height maps were acquired for ITO and for SAMs prepared at different blend ratios (Fig. S2; Table S2). Bare ITO exhibits the largest arithmetic mean roughness, S_a_ ≈ 1473 pm. Coating with single-component SAMs reduces S_a_ ≈ 862 pm for 2PACz and ≈ 893 pm for Me-4PACz; the slightly lower roughness of 2PACz indicates a marginally more uniform film, consistent with its smaller work function standard deviation relative to Me-4PACz. Blending 2PACz with Me-4PACz further decreases S_a_ ≈ 808 pm, implying improved coverage and lateral homogeneity. Consistently, 3D topographies show that the maximum peak-to-valley height range decreases from ≈ 15 nm on bare ITO to ≈ 12 nm after coating either single-component SAM and is smallest for the mixed SAM (≈ 10 nm), suggesting suppressed vertical aggregation.

Overall, introducing Me-4PACz into 2PACz can not only deepen the WF of the SAM mixture, but also results in a more uniform surface energy potential distribution than each individual SAM. Together, the smoother topography and improved WF homogeneity support enhanced interfacial contact and more efficient hole extraction at the perovskite/HTL interface.

### Characterization of the Perovskite Films

**Fig. 2:**
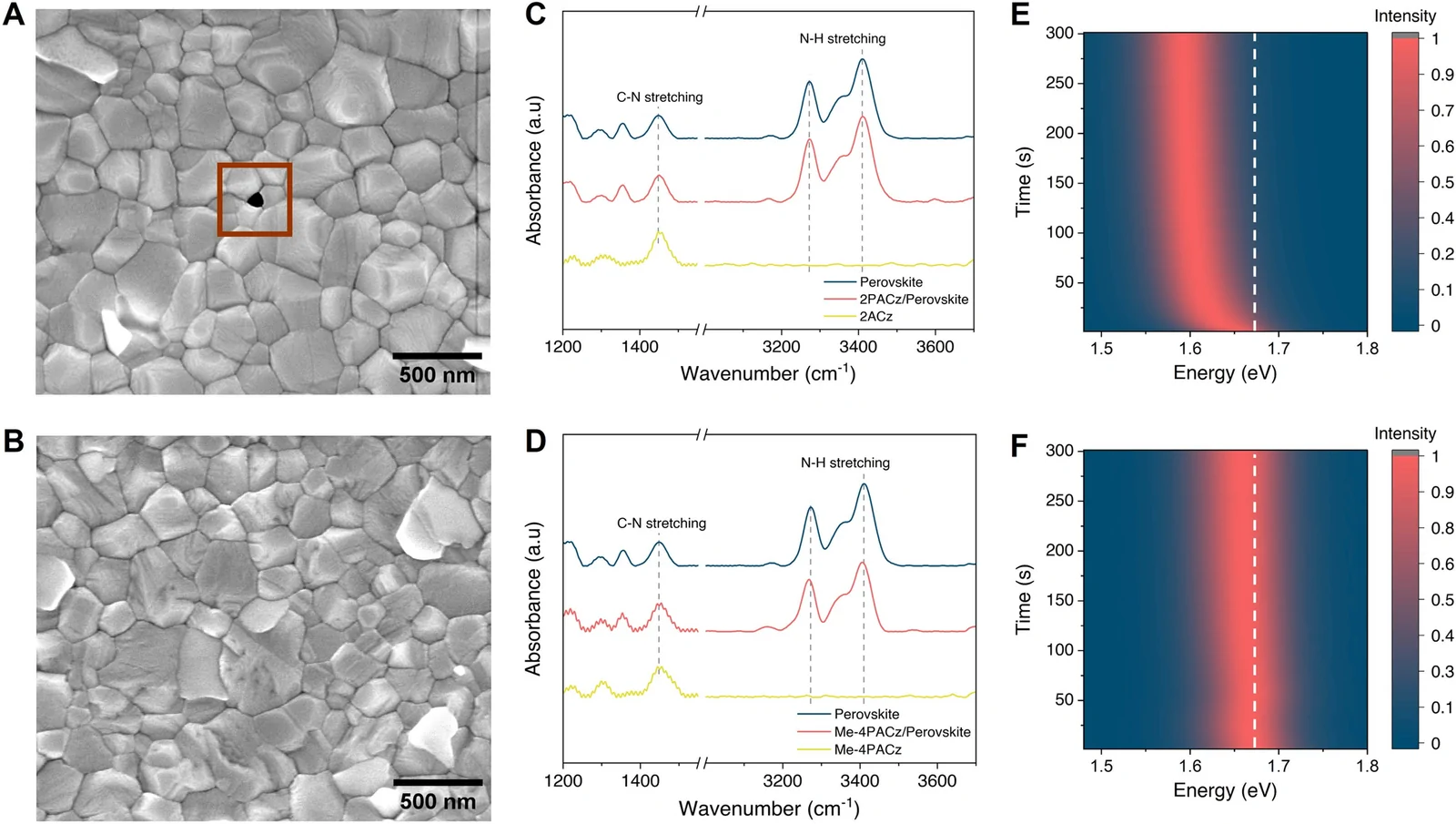
SEM images of the perovskite top surface using **A** 2PACz as HTL (The nanovoid was enclosed in a red square border) and **B** the mixture of 2PACz and Me-4PACz as the HTL. FTIR absorbance spectra of the perovskite films deposited on top of the **C** 2PACz as HTL and **D** Me-4PACz as the HTL. PL spectra scanning of the perovskite films using the **E** 2PACz as HTL and **F** the mixture of 2PACz and Me-4PACz as the HTL

As shown in Fig. S3, perovskite inks wet Me-4PACz poorly due to the hydrophobicity of its non-polar -CH_3_ termini, consistent with prior reports [53]. Several strategies have been reported to improve perovskite coverage, including evaporation-based methods [54] and solvent-assisted surface treatments [55], which enhance coverage by leaving surface-bound, high donor number species. In our study, blending Me-4PACz with 2PACz and increasing the 2PACz:Me-4PACz ratio improved wetting, enabling direct spin coating of perovskite without any additional surface treatment. To investigate the impact of the different HTLs on the perovskite film growth, SEM images of the perovskite layer were captured. The perovskite, with the composition [(Cs_0.22_FA_0.78_)_0.95_MA_0.05_]Pb[(Br_0.15_I_0.85_)_0.96_Cl_0.04_]_3_, was spin-coated onto 2PACz (control) and 2PACz:Me-4PACz mixture with ratio of 3:1 (target), separately. For the perovskite deposited on 2PACz, white PbI₂ particles were observed on the surface (Fig. 2a), as indicated by the PbI₂ peak at approximately 12.6° in the GIXRD pattern, which will be discussed in a later section. Additionally, nanovoids with an average diameter of approximately 144.6 ± 56.9 nm were observed (Figs. 2a and S4). The presence of voids can facilitate non-radiative recombination [56] and hinder efficient charge-carrier transport [57], which is consistent with previous reports [58]. The reduction in surface voids likely stems from decreased SAMs agglomeration [59], and high surface roughness [60], consistent with the lower surface roughness observed by AFM images. The formation of voids may also result from the segregation of the less volatile DMSO, which is commonly used with DMF to dissolve perovskite precursors. DMSO tends to become trapped within the film during deposition and escapes from the bottom, eventually leaving voids behind [57]. For the perovskite film deposited on the 2PACz:Me-4PACz mixture with a ratio of 3:1 (Fig. 2b), a pinhole-free perovskite film was achieved. No significant changes were observed in the perovskite grain size, which remains consistent for both the perovskite films spin coated onto 2PACz (251.0 ± 99.4 nm) and 2PACz&Me-4PACz (252.5 ± 98.9 nm) mixture HTLs (Fig. S5).

The crystallinity and grain size of the perovskite films on different HTLs were investigated using GIXRD. The GIXRD results indicate standard perovskite peaks at (001), (011), and (002) (Table S3; Fig. S6) at 2*θ* ≈ 14.2°, 20.1°, and 28.5°, respectively [27]. We fitted the diffraction peaks using Gaussian functions in OriginLab and observed that the peak intensities at (001), (011), and (002) planes increased significantly for perovskite films deposited on the mixture of 2PACz and Me-4PACz. Notably, the full width at half maximum (FWHM) decreased for (001) [by 92%], (011) [by 93%], and (002) [by 94%], respectively, indicating improved crystallinity and crystallite size. The intensity enhancement ratios for the (001) [by 116%] and (002) [by 110%] peaks were higher that of the (011) peak [by 104%], suggesting a stronger preferential orientation and larger crystallite size along the (001) and (002) planes, which is beneficial for efficient charge transport [61]. The PbI_2_ peak was observed on different SAMs-based perovskite films. Excess PbI_2_, in appropriate amounts (about 5%–10%) at surfaces and grain boundaries [62, 63], has been reported to be effective in passivating the defects, thereby enhancing the cell performance [64].

We then conducted FTIR measurements on the perovskite films deposited on 2PACz and Me-4PACz separately to investigate the distinct interactions between perovskite and each SAM. C–N stretching was observed at approximately 1448 cm^−1^, in line with previous work [65]. The peak position of the C–N stretching remained unchanged in perovskite films deposited on both 2PACz and Me-4PACz. N–H-related stretching was observed at wavenumbers around 3271 and 3409 cm^−1^, respectively, in the perovskite film with 2PACz as the underlayer, which is consistent with previous findings (Fig. 2c, d) [65]. The peak positions of N–H stretching, originating from formamidinium (FA⁺), exhibit redshifts of 3 and 5 cm^−1^ at 3271 and 3409 cm^−1^, respectively, in the perovskite film with Me-4PACz as the underlayer. Similar shifts (redshifts of 3 and 4 cm^−1^ at 3271 and 3409 cm^−1^, respectively) have also been observed in the mixture-based film (Fig. S7). This indicates an interaction between FA⁺ and Me-4PACz, where the aromatic ring in Me-4PACz acts as a hydrogen-bond acceptor for the N–H group in FA⁺ [66]. The methyl group (electron-donating group) on the aromatic ring increases the electron density of the aromatic ring through inductive effect which further enhances the bonding [67]. This enhanced interaction could passivate negatively charged vacancies (I^−^, Br^−^) at the interface [68]. A better passivation can reduce the recombination, which aligns with the higher implied *V*_oc_ (i*V*_oc_) indicated by the quasi-Fermi level splitting previously reported for perovskite films grown on Me-4PACz compared to those on 2PACz [54]. The previous study has shown that the hole-extraction flux via Me-4PACz to the conduction band of ITO can exceed that of 2PACz by more than an order of magnitude through enhanced band bending at the perovskite surface [39]. Recent research also highlights the mitigation of charge accumulation at the interface can improve the phase stability [16]. To investigate the difference of interaction between the Pb^2+^ and the 2PACz, and Me-4PACz, we conducted the XPS of perovskite films deposited on top of two different HTLs 2PACz and Me-4PACz. To probe the perovskite/HTL interfacial interaction, we intentionally thinned the perovskite layer by reducing the precursor concentration from 1.2 to 0.4 mmol L^−1^ and increasing the spin coating speed from 2000 to 6000 rpm. Cross-sectional SEM (Fig. S8) indicates an average film thickness of ≈ 44.2 ± 7.5 nm.

We observed two prominent peaks at ≈ 143.4 and ≈ 138.5 eV, corresponding to the Pb^2+^ 4*f*_5/2_ and Pb^2+^ 4*f*_7/2_ binding energy states, respectively, on the perovskite film deposited on 2PACz. A shift of approximately 0.06 and 0.08 eV towards lower energy was observed, for the peak positions Pb^2+^ 4*f*_5/2_ and Pb^2+^ 4*f*_7/2_, respectively, in the perovskite film deposited on the Me-4PACz. The decrease of the binding energy could be attributed to the chemical shielding effect caused by the added electron density, which reduces the nuclear attraction on core electrons, likely due to the stronger ability of lone pairs of electrons from the nitrogen atoms in 2PACz donating to Pb^2+^ ions. The stronger interaction between Pb^2+^ and Me-4PACz can explain the void-free nature of the perovskite film, as it reduces the trapping of DMSO within the film due to the competition between Me-4PACz and DMSO for Pb^2+^ binding. Meanwhile, obvious shoulder peaks at slightly lower binding energies (around 141.1 and 136.1 eV) correspond to metallic lead Pb^0^ 4*f*_5/2_ and Pb^0^ 4*f*_7/2_ (Fig. S9) were observed in the perovskite film deposited on the 2PACz, while the signal diminished in the 2PACz:Me-4PACz (3:1) blend and in the Me-4PACz-based perovskite films. The metallic lead has been reported to act as a recombination centre, deteriorating the device performance [69]. The signal of metallic Pb was significant reduced in the Me-4PACz-based perovskite film because of the stronger bonds compared to 2PACz with Pb^2+^ which could reduce metallic Pb through ion disproportionation [70].

To investigate the phase segregation of the perovskite film, we conducted photoluminescence measurements under continuous illumination (with spot diameter of ≈ 5.6 µm and power ≈ 22 µW) for 300 s. The perovskite film on 2PACz exhibited a significant redshift in bandgap energy, decreasing from 1.67 to 1.59 eV over 300 s (Fig. 2e), suggesting that halide phase segregation occurs post-illumination due to photogenerated carriers relaxing into low-energy I-rich states [71]. Charge accumulation plays a critical role in photo-induced halide segregation [72], as the localized charge carriers can generate an internal electric field that promotes ion migration [73]. In the latter part of this study, energy-level simulations revealed that appropriate interfacial energy alignment can eliminate undesirable energy barriers, thereby facilitating more efficient interfacial charge transfer. In contrast, the perovskite film on the mixture of 2PACz and Me-4PACz shows minimal bandgap shift, dropping only slightly from 1.67 to 1.66 eV under the same illumination conditions (Fig. 2f). Space charge-limited current (SCLC), a standard probe of bulk and interfacial traps, is employed to investigate the trap-state density at the perovskite/HTL interface. We employed an HTL-only structure of ITO/SAMs/perovskite/ 4-Butyl-N,N-diphenylaniline homopolymer, Poly(4-butyl-N,N-diphenylaniline), Poly(4-butyltriphenylamine), and Poly[N,N′-bis(4-butylphenyl)-N,N′-bis(phenyl)-benzidine] (Poly-TPD)/Ag (Fig. S10).We use our devices differ only at the perovskite/HTL interface and surface defects usually exceed bulk defects [68, 74], rendering the signal interface-dominated.

According to Eq. 1:

1 $$N_{{\mathrm{t}}} = \frac{{2\varepsilon_{0} \varepsilon V_{{{\mathrm{TFL}}}} }}{{eL^{2} }}$$

Here *N*_t_ is the trap-state density, and *ε*_*0*_ denotes the vacuum permittivity. *e* represents the relative dielectric constant of FAPbI_3_ (*ε* = 46.9) [75]. *e* is the elementary charge is the electron charge, and *L* is the thickness of the perovskite film (≈ 400 nm, as shown in Fig. S5).

The trap-state density (*N*_t_) can be calculated using the trap-filled limit voltage (*V*_TFL_). The *V*_TFL_ values for devices using 2PACz and a mixture of 2PACz:Me-4PACz were determined to be 0.8 and 0.69 V, respectively. The *N*_t_ for the control device was calculated to be 2.62 × 10^16^ cm^−3^, which was reduced to 2.24 × 10^16^ cm^−3^ when using the 2PACz:Me-4PACz HTL. This represents a 14.8% drop in the trap-state density.

### Performance of the Perovskite Single Junction Solar Cells

**Fig. 3:**
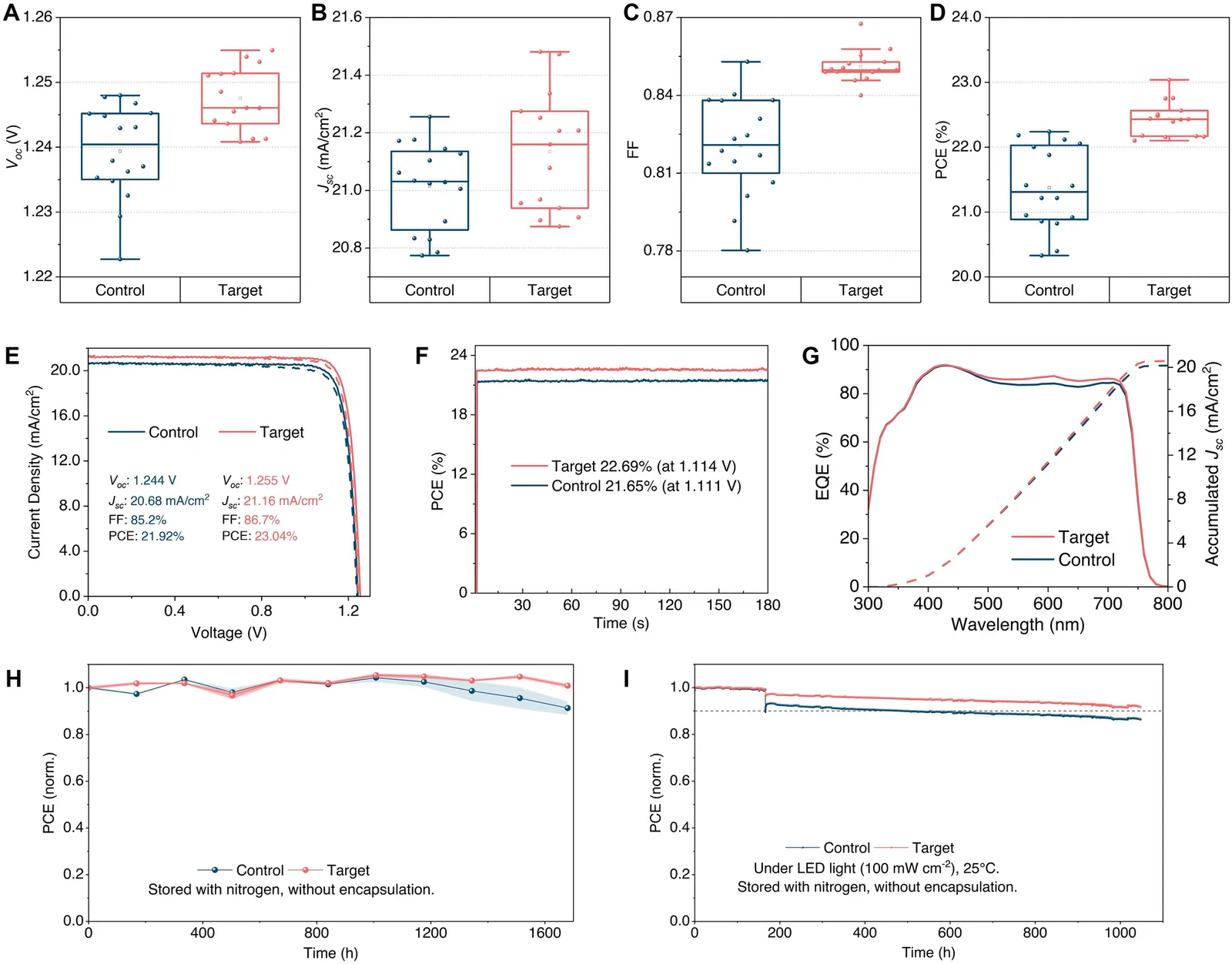
Photovoltaic performance parameters: **A** *V*_oc_, **B** *J*_sc_, **C** FF, and **D** PCE of PSCs using 2PACz (control) or mixture of 2PACz:Me-4PACz (target) as HTL. **E** *J*–*V* curves of PSCs using 2PACz (control) or mixture of 2PACz:Me-4PACz (target) as HTL. **F** Steady-state efficiency under simulated AM 1.5G illumination. **G** EQE spectra with integrated *J*_sc_ of PSCs. **H** Shelf-stability test with five samples for each condition, the lines represent the averaged measured value, while the shaded area is the standard deviation for five samples. **I** Light stability test under continuous 1-sun light illumination

We then fabricated inverted PSCs based on different SAMs with the structure ITO/SAMs/perovskite/PDAI_2_/C60/SnO_*x*_/Ag. The perovskite composition is [(Cs_0.22_FA_0.78_)_0.95_MA_0.05_]Pb[(Br_0.15_I_0.85_)_0.96_Cl_0.04_]_3_, with a bandgap of 1.67 eV. The cross-sectional SEM (Fig. S11) shows continuous layers with thickness ≈ 400 nm, C60 ≈ 16 nm, and SnO_x_ 13 nm, no voids or delamination were observed at the HTL/perovskite interface. The control champion 2PACz-based device achieved a PCE of 21.92% (Fig. 3b), with a steady-state efficiency of 21.65% (Fig. 3d). The analysis of device performance statistics shows a significant improvement in PCE for device based on blended SAM, thanks to an increase in all photovoltaic metrics including *V*_oc_, FF, and *J*_sc_ (Fig. 3a–d). As the percentage of the Me-4PACz increases, the *V*_oc_ improves till the ratio reaches 4:1, after which it starts to decrease. The *J*_sc_ increases as the percentage of the Me-4PACz increases up to a ratio of 5:1. The FF has a trend of increase as the percentage of the Me-4PACz starts to increase. The ratio of 2PACz to Me-4PACz was optimized at 3:1 (Fig. S12), leading to a *V*_oc_ of 1.255 V, a FF of 86.7%, and a *J*_sc_ of 21.16 mA cm^−2^, and a PCE of 23.04%, with the stabilized PCEs reaching 22.69% (Fig. 3d). The FF, with only a 4.15% deficit (Fig. S13; Table S1), is the highest reported value in WBG PSCs to date. This FF deficit for the first time is smaller than that reported for low bandgap perovskite solar cells [14]. The series resistance (*R*_s_) for the target devices is calculated to be 2.05 Ω cm^2^ as compared with 2.22 Ω cm^2^ for the control devices. There is an improvement in *J*_sc_ by 0.4 mA cm^−2^ in 2PACz:Me-4PACz mixture HTL-based PSC as compared with the control device, consistently calculated from both *J–V* and EQE measurements. The integrated *J*_sc_ of 20.17 mA cm^−2^ and 20.58 mA cm^−2^ for the control and target devices, respectively, derived from EQE measurement matches well with those from *J–V* curves. The EQE spectra (Fig. 3g) of the mixed SAM device show consistently higher response across 370–800 nm. The modest uplift for the target device and the sharper red-edge indicate reduced interfacial recombination, as reflected by the smaller Urbach energy [76, 77] (≈ 24.8 meV) compared with the control device (≈ 23.73 meV) (Fig. S14). Given that the perovskite thickness was approximately 400 nm, we further investigated the effect of increased thickness on device performance by fabricating cells by reducing the spin coating rate. The thicker device (perovskite thickness ≈ 1143 nm suggested by the cross-sectional SEM in Fig. S15) exhibited an enhancement in *J*_sc_ by ≈ 0.8 mA cm^−2^; however, both FF and *V*_oc_ declined by ≈ 9.3% and ≈ 30 mV (Table S4), respectively, attributable to increased defect density and bulk charge recombination [78, 79]. As a result, the overall PCE decreased by ≈ 3.2%. The increase in *J*_sc_ suggests that further optimization of the perovskite thickness could potentially enhance the PCE. Because the HTL/perovskite interface governs both *V*_oc_ and FF, we quantified the quasi-Fermi level splitting (QFLS) to investigate the losses [80–83]. As summarized in Fig. S16 and Table S5, the SQ radiative limit for 1.67 eV bandgap is *V*_oc_ = 1.37 V and FF = 90.8%. The device measured by 1-sun *J–V* yields *V*_oc_ = 1.252 V and FF = 85.6%; Suns-PL (1.259 V) and Suns-*V*_oc_ (1.251 V) closely match the *J–V* value, indicating negligible transport penalties. The total voltage deficit relative to SQ is 118 mV, of which 111 mV stems from non-radiative recombination (*ΔV*_non-radiative_ = SQ—Suns-PL) while only 7 mV is attributable to transport/extraction (Suns-PL—*J–V*). The 5.2% FF deficit decomposes into 3.7% associated with recombination/diode quality (SQ—Suns-*V*_oc_) and 1.5% due to transport/contact effects (Suns-*V*_oc_—*J–V*). Collectively, these results indicate a transport-robust device whose remaining headroom is dominated by non-radiative losses; therefore, further gains should prioritize raising QFLS via targeted interfacial and bulk passivation (and reducing optical parasitic) [84], with only modest improvement expected from additional transport optimization (about 1%–2% of FF).

Moreover, we evaluated a bilayer strategy by sequentially spin coating 2PACz and Me-4PACz in different orders, with the rationale that the second SAM layer could mitigate desorption of the underlying layer and thereby enhance interfacial contact [85–87]. As shown in Fig. S17, perovskite films exhibited good coverage on the 2PACz/Me-4PACz bilayer, whereas poor coverage was observed on Me-4PACz/2PACz, likely due to the predominance of hydrophobic Me-4PACz at the ITO surface. Consistently, Fig. S18 and Table S6 demonstrate that the 2PACz/Me-4PACz bilayer improved device performance relative to single-layer 2PACz, with the FF increasing from 82% to 85% and hysteresis decreasing from 7.6% to 6.5%. These enhancements can be attributed to improved interfacial contact, reduced desorption, and the formation of an energy-level cascade at the interface [88]. Notably, the mixed SAM outperformed the bilayer 2PACz/Me-4PACz, yielding further improvements in *V*_oc_ (1.27–1.28 V), FF (85%–86%), and a pronounced reduction in hysteresis (6.5%–3.6%). This additional improvement is likely due to the reduced number of interfacial layers combined with tailored interfacial energy-level alignment. To evaluate the applicability of our interface strategy, we fabricated devices with a 1.73 eV Rb-FA_0.75_MA_0.15_Cs_0.1_PbI_2_Br perovskite [37]. As shown in Fig. S19 and Table S7, the mixed SAM outperformed the pure 2PACz, yielding further improvements in *V*_oc_ (≈ 10 mV), *J*_sc_ (≈ 0.27 mA cm^−2^), FF (≈ 6.6%), PCE (≈ 2%, absolute), and a pronounced reduction in hysteresis (9.12–5.85%). We attribute these gains to the deeper valence-band maximum of the 1.73 eV composition, for which a higher and more laterally uniform HTL work function provided by the blended SAM better matches the perovskite VBM, lowering the hole-extraction barrier, suppressing interfacial recombination, and thereby mitigating hysteresis.

To further improve the cell efficiency, we optimized the concentration of PDAI_2_ used as the top surface passivation layer and replaced ITO with FTO to increase the *J*_sc_. The intrinsically rougher FTO surface enhances light trapping via increased forward scattering, thereby reducing optical losses and improving absorption in the perovskite layer [31]. This optimization resulted in improved *J–V* performance, resulting in the cell performance with *V*_oc_ of 1.268 V, *J*_sc_ of 22.4 mA cm^−2^, FF of 84.7%, and a PCE of 24.07%, with stabilized PCE of 23.47% (Fig. S20). The cell was sent to Commonwealth Scientific and Industrial Research Organisation (CSIRO) for certification, achieving a certified result of 23.42% with FF of 86.8% (4.1% deficit) and a *J*_sc_ of 21.7 mA cm^−2^ (Fig. S21).

We then tested the long-term stability of the PSCs. We investigated the dark stability of unencapsulated PSCs with different HTL layers, 2PACz and 2PACz&Me-4PACz (Fig. 3h) separately, in a nitrogen atmosphere at ambient temperature. After 1680 h of storage, the average PCE of the control devices decreased to 91% of their initial efficiency, whereas the target devices maintained 100% of their original PCE. To further assess operational stability, unencapsulated devices were exposed to continuous 1-sun equivalent illumination using a UV filter glass and biased at the around maximum power point (MPP) voltage in a nitrogen atmosphere at 25 °C (Fig. 3i). During the initial 166 h, both devices exhibited comparable stability. Subsequently, a system error subjected the devices to more severe conditions—open-circuit operation under illumination for 1 day. After rectifying the system and restoring standard conditions, the target device retained 97% of its original performance, whereas the control device maintained 93%. After 1050 h of tracking, the PCE of the control devices declined to 85.5% of their initial value, whereas the target devices retained 90.5% of their original PCE.

### Performance Loss Analysis and the Simulation

To understand the mechanism for the cell performance improvement, we first quantified the recombination loss at the perovskite/HTL interface. Light intensity-dependent *V*_oc_ can be used to calculate the ideality factor, which diagnoses recombination pathways in PSCs. Lower ideality factor generally signifies suppressed Shockley–Read–Hall recombination in the perovskite bulk and at interfaces [85, 86], so *V*_oc_ intensity analysis quantifies recombination losses. Consistent with the SCLC analysis, our devices differ only at the perovskite/HTL interface and, because surface defects typically exceed bulk defects [68, 74], the extracted ideality factor is predominantly interface-dominated. The light intensity-dependent *V*_oc_ was measured in solar cell devices using 2PACz and a 2PACz:Me-4PACz mixture as HTLs, respectively. According to the relationship between the *V*_oc_ and light intensity (Eq. 2):

2 $$V_{{{\mathrm{oc}}}} = \frac{kT}{q}{\mathrm{ln}}\left( {\frac{I}{{I_{0} }}} \right){ }$$

Here *k* is the Boltzmann constant, *T* is the absolute temperature, *q* denotes the elementary charge, *I* is the light intensity, and *I*_*0*_ is the reverse saturation current, which is influenced by recombination mechanisms.

In an ideal scenario dominated by radiative recombination, the slope of the *V*_oc_ versus ln(*I*) should be approximately to the value of (*kT/q*), around 59.6 mV per decade increase in light intensity at 25 °C [89]. The extracted ideality factor value for the control sample is 2.28 [90], which is higher than that of the 2PACz:Me-4PACz HTL device at 2.01 (Fig. S22 and Table S8), indicating a higher defect assisted recombination process occurring at the perovskite/HTL interface [91].

**Fig. 4:**
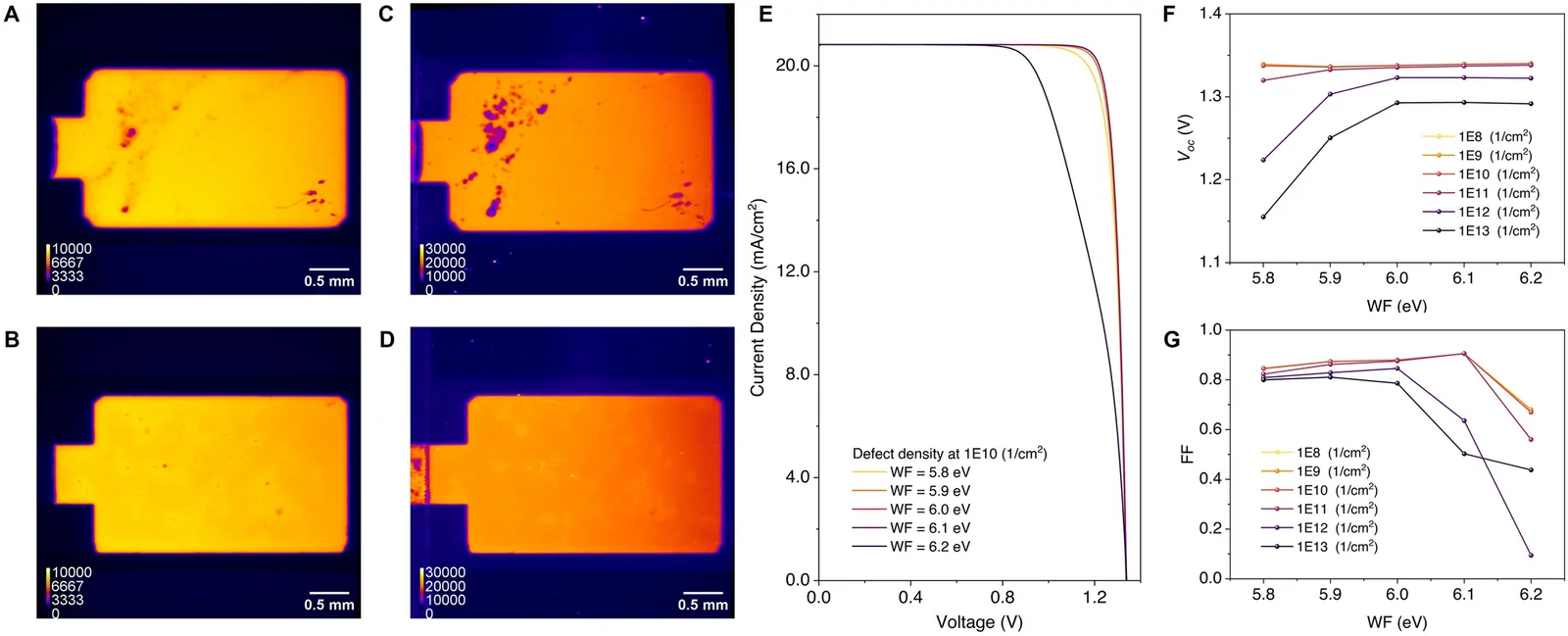
EL images of the perovskite solar cell devices using **A** 2PACz as HTL and **B** the mixture of 2PACz and Me-4PACz as the HTL. PL images of the perovskite solar cell devices using the** C** 2PACz as HTL and **D** the mixture of 2PACz and Me-4PACz as the HTL. **E** Simulated results of the IV curves with influence of the work function of the HTL with HTL/perovskite interface defect density at 1E10 (1/cm^2^). Simulated results showing the influence of the WF of the HTL on **F** *V*_oc_, and **G** FF of the simulated PSCs under different interface defects concentration

We then conducted EL and PL mapping tests to assess the homogeneity of the film, as shown in Fig. 4a–d. The perovskite film deposited on 2PACz exhibited inhomogeneity across the active area, with the PL image indicating recombination loss sites distributed on the film. The inhomogeneity was also observed in the EL image, suggesting poor contact of the perovskite with the charge transport layer within this region, which could be a recombination site [92]. The EL and PL mapping of the target devices (Fig. 4a–d) show superior uniformity compared to the control film, suggesting that the issue originated at the HTL/perovskite interface, given that the top structure beyond the perovskite remained the same. Overall, the perovskite films on the 2PACz:Me-4PACz mixture exhibited reduced non-radiative recombination and enhanced uniformity, making them well-suited for scale-up fabrication.

In our experimental results, we have two further observations that require explanation: (1) The improvement of PCE is dominated by increase in *V*_oc_ and FF, and (2) the percentage of the relatively increased FF (3.7%) is larger than the relatively increased percentage of *V*_oc_ (0.7%). This prompts a systematic investigation into the effect of composition engineering of HTL on the *V*_oc_ and FF of the whole device. Our hypothesis is that varying defect density at the HTL/perovskite interface can cause different trap-assisted recombination at the interface, which will re-distribute the electric field at the interface, changing the band alignment, and modulating the carrier transport. Based on the drift diffusion model of carriers, we simulated the iPSC structure using COMSOL software (Table S9) and found that the improvement in photovoltaic performance was attributed to a reduction in the energy barrier at the HTL/perovskite interface (Figs. S23, S24, S25). At a lower WF of 5.8 eV of the HTL, an energy barrier was observed in the Valence Band (VB), indicating restricted hole injection from the perovskite into the ITO. This barrier disappeared as the absolute WF increased to 5.9 eV. However, as the WF continues to increase, a valley emerged at the HTL/perovskite interface, facilitating hole transport. Yet, when the WF reached 6.2 eV, the valley deepened, potentially trapping carriers at the interface. We then further elaborate the study into the influence of HTL/perovskite defect density ranging from 1 × 10^8^ to 1 × 10^13^ cm^−2^. Figure 4f indicates the impact of defect density on the device *V*_oc_. At lower defect density (1 × 10^8^–1 × 10^10^ cm^−2^), *V*_oc_ firstly decreases as the WF increases from 5.8 to 5.9 eV and continues increasing as the WF further increased. When the defect density increases to 1 × 10^11^ cm^−2^, the *V*_oc_ increases as the WF increases. For higher defect densities (1 × 10^10^–1 × 10^13^ cm^−2^), the *V*_oc_ increases with increasing WF up to 6.0 eV, after which it starts to decline. The optimal WF for achieving the *V*_oc_ highest is around 5.9–6.0 eV. As defect density increases, *V*_oc_ decreases significantly, particularly for higher WF values (over 6.1 eV), due to intensified non-radiative recombination at the interface. The impact of modifying the WF from 5.8 to 6.0 eV on increasing *V*_oc_ becomes more significant as the defect density increases. Figure 4g indicates the impact of defect density on the device FF. At lower defect density (1 × 10^8^–1 × 10^11^ cm^−2^), FF firstly increases as the WF increases from 5.8 to 6.1 eV and starts to drop as the WF further increased. For higher defect densities (1 × 10^12^–1 × 10^13^ cm^−2^), the FF increases with increasing WF up to 6.0 and 5.9 eV, respectively, after which it starts to decline. The optimal WF for achieving the *V*_oc_ highest is around 6.0–6.1 eV. As defect density increases, FF decreases significantly. The impact of modifying the WF from 5.8 to 6.1 eV on improving the FF becomes more pronounced as the defect density decreases. Our observations indicate that the defect density at the HTL/perovskite interface plays a critical role in how the WF influences *V*_*oc*_ and FF. At lower defect densities, the improvement in FF contributes more significantly to the overall device performance, whereas at higher defect densities, the enhancement in *V*_*oc*_ becomes the dominant factor.

### Semi-Transparent and 4-T Tandem Perovskite/Si Solar Cell

**Fig. 5:**
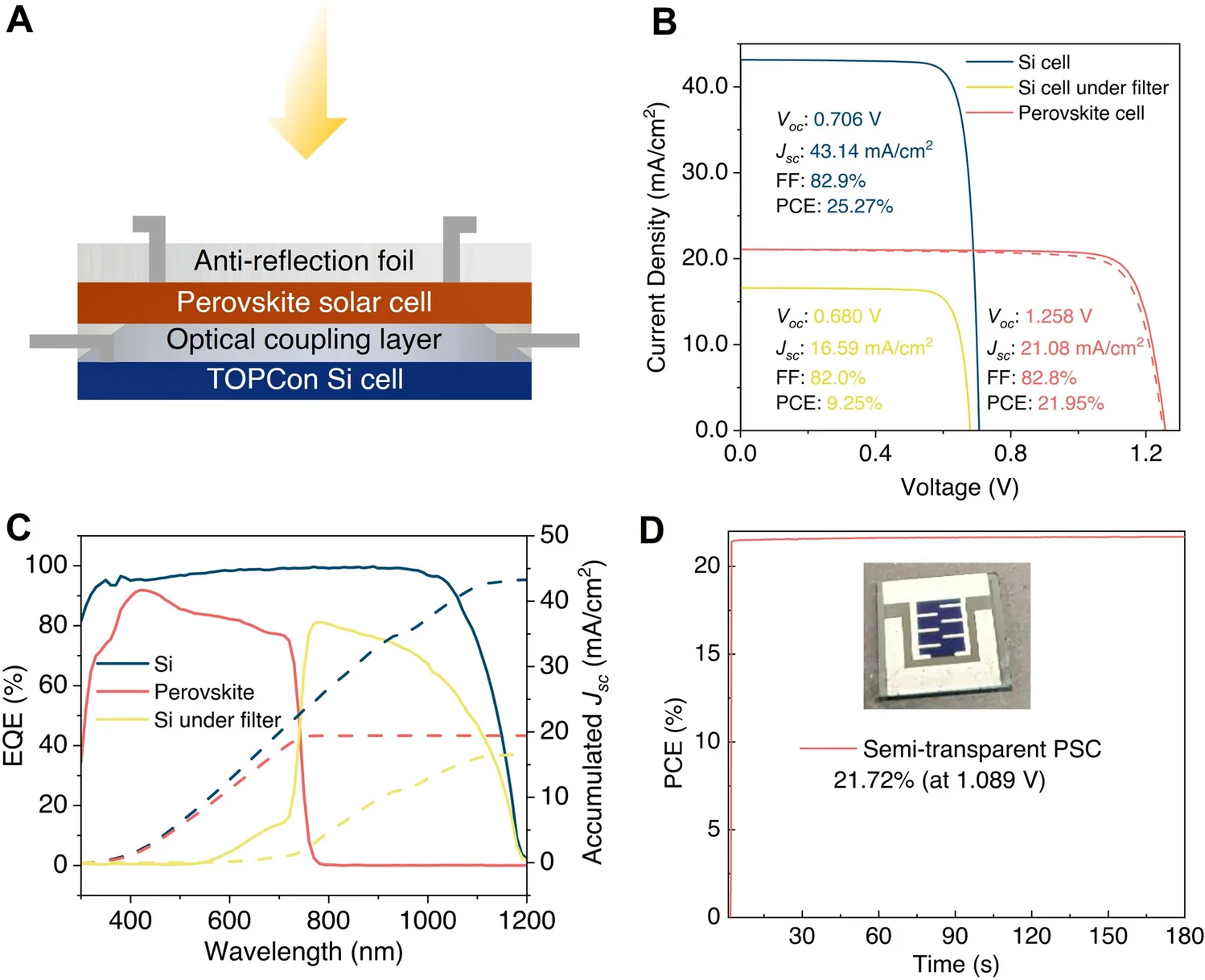
**A** Schematic structure design of the **4-T** perovskite/Si tandem solar cell. **B** *J–V* curves of Si solar cell with (blue) and without filter (yellow), and the semi-transparent perovskite solar cell (red). **C** EQE spectra with integrated *J*_sc_ of the TOPCon Si solar cell (blue), the semi-transparent PSC (red), and the TOPCon Si solar cell with perovskite filter atop (yellow). **D** Steady-state efficiency of the semi-transparent perovskite solar cell under simulated AM 1.5G illumination

The optimized 1.67 eV perovskite PSCs is combined with the tunnel oxide passivated contact (TOPCon) Si solar cell for an efficient 4-T tandem solar cell [93]. The 4-terminal configuration, free from the constraints of current matching, allowed for less stringent bandgap optimization [94], and the direct enhancement of the top semi-transparent perovskite layer can significantly boost the overall efficiency of the tandem solar cell. Figure 5a illustrates a schematic of the 4-T mechanically stacked perovskite/Si tandem solar cell. To enhance the light absorption, an anti-reflection foil [38], consisting of textured silicone with inverted pyramids, was applied to the glass side of the semi-transparent PSCs. Additionally, a refractive index-matching layer (n_D_ = 1.414 ± 0.002 at 25 °C) was inserted between the two cells [94]. We then employed the efficient 2PACz:Me-4PACz mixture HTL for an efficient wide-bandgap, semi-transparent perovskite solar cell, using the structure glass/ITO/2PACz:Me-4PACz/perovskite/PDAI_2_/C60/SnO_x_/IZO/Ag fingers. Figure 5b presents the performance of each sub-cell, with a summary provided in Table 1. The champion semi-transparent perovskite top cell demonstrated an efficiency of 21.21% in forward scanning (FS) and 21.95% in reverse scanning (RS), with a *V*_oc_ of 1.258 V, *J*_sc_ of 21.08 mA cm^−^2, and FF of 82.8%, which is one of the highest semi-transparent cells of 1.67 eV reported [10]. The stabilized PCE is 21.72%. When filtered through a semi-transparent perovskite cell with the similar layer structure, a Si cell with an efficiency of 25.27% under full spectrum maintained with *V*_oc_ of 0.68 V, *J*_sc_ of 16.59 mA cm^−^2, FF of 82.0%, and a PCE of 9.25%. The EQE spectra (Fig. 5c) for the Si cell, the semi-transparent perovskite cell (transmittance spectra in Fig. S26), and the Si cell under the perovskite filter are displayed in Fig. 5d. We noticed the Si sub-cell EQE remained below 80% across 800–1200 nm, indicating substantial room for improvement. Further gains will require optical engineering to reduce reflection (e.g. optimizing the refractive index-matching interlayer and anti-reflection coating, exploring different types of Si cells with less shading) and to increase top cell transmittance, for example by thinning the TCO (e.g. IZO) layer. The structure of the Si solar cell is illustrated in (Fig. S27). The assessment of tandem stability involves evaluating both the semi-transparent top perovskite solar cell and the bottom silicon solar cell. As previously reported, silicon solar cells exhibit significantly higher stability compared to perovskite devices [95–98]. Therefore, we focused on investigating the light stability of the semi-transparent perovskite cell. We evaluated both the photovoltaic performance and optical transmittance of the semi-transparent perovskite cells after continuous illumination, as the latter will have a direct impact on the photocurrent of the bottom silicon cell in the tandem configuration. We exposed unencapsulated semi-transparent devices, similar to the one used in the tandem configuration, to continuous 1-sun equivalent illumination (with UV filter glass) under nitrogen at 25 °C, biased near the MPP voltage (Fig. S28). Over 430 h, devices with blended SAMs retained 72.4% of their initial PCE, whereas those with pure 2PACz retained 65.4%. The degradation was faster than in opaque devices, which is likely caused by an accelerated loss between 40 and 100 h, due to accidental falling off of the UV filter glass. It has been reported that UV exposure promotes C–N bond splitting in carbazole-based SAMs [97, 98]. Parameter evolution indicates that performance losses were dominated by decreases in *J*_sc_ and FF: For 2PACz, *J*_sc_ decreased from 21.21 to 18.96 mA cm^−2^ and FF from 81 to 59%; for the blended SAMs, *J*_sc_ decreased from 21.38 to 19.29 mA cm^−2^ and FF from 85% to 68%. We attribute the improved retention with blended SAMs to a more homogeneous HTL surface, improved perovskite coverage, and enhanced interfacial carrier extraction. In addition (Fig. S26), the blended SAM devices exhibited only negligible spectral transmittance changes (≈ ± 2.5%): increases at 800–980 and 1100–1200 nm, and a slight decrease at 980–1100 nm, implying minimal impact on absorption in the Si bottom cell. Overall, the efficiency of the 4-terminal perovskite/Si tandem solar cell reaches 30.97%, making it among the highest reported value for this TOPCon-based perovskite/Si tandem device utilizing industrially dominant Si technology (Table S10).

**Table 1:** Photovoltaic parameters of 4-T perovskite/silicon tandem solar cells

	Area [cm^2^]	*V*_oc_ [V]	*J*_sc_ [mA cm^−2^]	FF	PCE [%]
Si cell	4	0.706	43.14	82.9	25.27
Si cell under perovskite filter	4	0.680	16.59	82.0	9.25
ST perovskite cell–RS	0.35	1.258	21.08	0.828	21.95
ST perovskite cell–FS		1.251	21.10	0.803	21.21
ST perovskite cell–steady-state output					21.72
Tandem cell					30.97

## Conclusions

In conclusion, an HTL/perovskite interface energy alignment strategy was employed to enhance the photovoltaic performance of WBG iPSCs. The hydrophobic Me-4PACz was mixed with the 2PACz to overcome the wettability issue, increasing the perovskite film coverage. A WF increase of 1.14 eV was observed on the ITO surface modified with the 2PACz&Me-4PACz mixture, compared to a 0.72 eV increase with 2PACz alone. This significant WF enhancement effectively reduces the energy barrier at the HTL/perovskite interface, as demonstrated by COMSOL simulation results, facilitating charge transfer and enhancing overall device performance. The introduction of Me-4PACz mitigated the hole transport energy barrier at the HTL/perovskite interface and enhanced hole extraction. The mixture-based approach provided uniform bottom HTL energy distribution and perovskite film coverage, as confirmed by PL and EL imaging, which is crucial for reducing shunting effects and advancing commercialization. FTIR analysis revealed interactions between the FA⁺ cation and the methyl-substituted aromatic ring in Me-4PACz, which contributes to the passivation of recombination losses caused by negatively charged vacancies in the perovskite, thereby improving the *V*_oc_. This resulted in a record FF 86.8% for the WBG iPSCs, with a certified PCE of 23.42%. Device simulations demonstrate that proper energy alignment at the HTL/perovskite interface removes the energy barrier, significantly enhancing solar cell performance. This improvement contributed to better phase stability due to the enhanced charge extraction at the interface. Unencapsulated devices demonstrated excellent long-term stability, retaining over 90% of their initial PCE after 1000 h of MPP tracking and showing no efficiency loss after 1680 h of dark storage testing. When combined with a bottom silicon cell based on industrial dominant TOPCon configuration, the 4-T tandem devices achieved a PCE of 30.97%, one of the highest efficiencies reported for 4-T perovskite/Si (TOPCon) tandem solar cells. The HTL/perovskite interface energy alignment strategy addresses the challenge of simultaneously maintaining high FF and *V*_oc_ in perovskite solar cells, achieving a state-of-the-art PCE for WBG iPSCs with a bandgap of 1.67 eV. This advancement provides insights into developing strategies to further enhance device efficiency in future.

## Supplementary Information

Below is the link to the electronic supplementary material.

Supplementary file1 (DOCX 36.2 MB)

Supplementary file2 (CSV 232 KB)

Supplementary file3 (CSV 343 KB)
