# Supplementary material for: High-Efficiency Perovskite/Silicon Tandem Solar Cells Based on Wide-Bandgap Perovskite Solar Cells with Unprecedented Fill Factor
Source: Nanomicro Lett. 2026 Jan 14;18:122. doi: 10.1007/s40820-025-01959-y (PMC12799847; doi:10.1007/s40820-025-01959-y)
Supplement: Supplementary file 1 — Supplementary file1 (DOCX 36.2 MB) [file 40820_2025_1959_MOESM1_ESM.docx]

Supporting Information for

**High-Efficiency Perovskite/Silicon Tandem Solar Cells Based on Wide-Bandgap Perovskite Solar Cells with Unprecedented Fill Factor**

Li-Chun Chang^1^, The Duong^1^, Viqar Ahmad^1^, Hualin Zhan^1^, Anh Dinh Bui^1^, Jana-Isabelle Polzin^2^, Armin Richter^2^, Gabriel Bartholazzi^1^, Keqing Huang^1^, Zhongshu Yang^1^, Wei Wang^1^, Yihui Hou^1^, Li Li^3^, Qian Cui^1^, Rabin Basnet^1^, Jianfei Yang^4^, Hong Lin^4^, Guozheng Du^1^, Khoa Nguyen^1^, Dang-Thuan Nguyen^1^, Lachlan E. Black^1^, Daniel MacDonald^1^, Daniel Walter^1^, Klaus J. Weber^1^, Kylie R. Catchpole^1^, Heping Shen ^1,^ *

^1^ School of Engineering, The Australian National University, Canberra, Australian Capital Territory, 2601 Australia

^2^ Division Photovoltaics, Fraunhofer Institute for Solar Energy Systems ISE, Heidenhofstrasse 2, 79110 Freiburg, Germany

^3^ Research School of Physics, The Australian National University, Canberra, Australian Capital Territory, 2601 Australia

^4^ State Key Laboratory of New Ceramics & Fine Processing, School of Materials Science and Engineering, Tsinghua University, Beijing 100084, P. R. China

*Corresponding author. E-mail: [Heping.Shen@anu.edu.au](mailto:Heping.Shen@anu.edu.au) (Heping Shen)

# S1 Supplementary Notes

## S1.1 Cells fabrication process

**Si solar cells:**

The Tunnel Oxide Passivated Contact (TOPCon) solar cells fabricated within this study have a designated area of 2×2 cm^2^ and were made of high-quality (100)-oriented n-type float-zone (FZ) Si wafers with a diameter of 100 mm. The thickness of the wafer was 400 µm. The front side of the solar cells features an alkaline textured (random-pyramids) surface with a boron-doped p^+^ emitter. The emitter was formed by BBr_3_ tube furnace diffusion at 870 °C followed by drive-in oxidation, resulting in an emitter with a sheet resistance of *R*_sheet,p+_= 300 Ω/sq. The emitter was passivated with a 10 nm thick Al_2_O_3_ layer deposited with plasma-assisted atomic layer deposition [S1]. The front side was additionally coated with a double-layer antireflection coating (DARC) consisting of 50 nm SiN_x_ grown by plasma-enhanced chemical vapor deposition (PECVD) and 90 nm thermally evaporated MgF_2_. Prior to the MgF_2_ deposition, contact openings were etched wet-chemically through the front side Al_2_O_3_/SiN_x_ coating and the front side metal contacts were formed with a stack of e-beam evaporated Ti/Pd/Ag (833 µm pitch). To reduce surface recombination losses at the front metal contacts, a heavily doped p^++^ emitter is located underneath the contacts, also diffused in a BBr_3_ tube furnace process and a subsequent drive-in. All structuring processes required for the front side are based on photolithography (partially to structure thermally grown SiO_2_ mask layers) and the lift-off technique. The full-area TOPCon electron contact on the rear surface is formed by an ultra-thin wet-chemical (HNO_3_-based) SiO_x_ layer covered by a 15 nm thick phosphorous-doped Si compound layer deposited with PECVD, which is annealed afterwards in a tube furnace at 800 °C (process details in [S2, S3]). The rear side metallization is realized by thermally evaporated Ag.

**Opaque and semitransparent perovskite solar cells with 1.67 and 1.73 eV:**

To prepare the indium tin oxide (ITO) or fluorine-doped tin oxide (FTO) substrate for the perovskite solar cell, the first step involved ultrasonic cleaning using soap water, acetone, 2-propanol, ethanol, and deionized water, and treated by UV-Ozone for 15 min after the cleaning process. The self-assembled monolayers (SAMs) and the perovskite layer were spin-coated on to the ITO substrates at room temperature in a N_2_ filled glovebox. The SAMs solution with different ratio of [((2-(9H-carbazol-9-yl)ethyl)phosphonic acid) (2PACz), and 4-(3,6-dimethyl-9H-carbazol-9-yl) butyl) phosphonic acid (Me-4PACz)] with a concentration of 1.7 mmol/L in ethanol [S4] was spun on the ITO substrate at 3000 rpm with a ramp of 3000 rpm/s for 30 s. The substrate was subsequently annealed on a hot plate at 100 °C for 10 min.

Subsequently, **A)** the 1.67 eV perovskite film was deposited on the substrate by one-step spin-coating using a solution consisting of [(Cs_0.22_FA_0.78_)_0.95_MA_0.05_]Pb[(Br_0.15_I_0.85_)_0.96_Cl_0.04_]_3_, with a total of 1.2 mmol (68.6 mg CsI, 99.1 mg PbBr_2_, 161 mg FAI, 429 mg PbI_2_) in 0.8 ml DMF and 0.2 ml DMSO. This solution also contained 5 mol% MA-Cl and 3 mol% PbCl_2_ additives. The perovskite precursor solution was spin-coated on the substrate at 2000 rpm with a ramp of 1000 rpm/s for 12 s. **B)** the 1.73 eV perovskite film was deposited on the substrate by one-step spin-coating using a solution consisting of Rb-FA_0.75_MA_0.15_Cs_0.1_PbI_2_Br. First, prepare four 1-mL precursor stocks. For FAPbI_2_Br, dissolve FAI (149.6 mg, 0.87 M), FABr (53.8 mg, 0.43 M), PbI_2_ (441.2 mg, 0.957 M) and PbBr_2_ (157.8 mg, 0.43 M) in DMF: DMSO = 4:1 (v/v). For MAPbI_2_Br, dissolve MAI (138.2 mg, 0.87 M), MABr (48.2 mg, 0.43 M), PbI_2_ (441.2 mg, 0.957 M) and PbBr_2_ (157.8 mg, 0.43 M) in DMF: DMSO = 4:1 (v/v). For CsPbI_2_Br, dissolve CsI (226.2 mg, 0.87 M), CsBr (91.6 mg, 0.43 M), PbI₂ (441.2 mg, 0.957 M) and PbBr_2_ (157.8 mg, 0.43 M) in DMSO, then heat at 80 °C for 30 min to fully dissolve. For RbI, dissolve RbI (275.6 mg, 1.3 M) in DMSO. Finally, mix the four stocks in the following volumes to obtain the working perovskite ink: 750 µL FAPbI_2_Br + 150 µL MAPbI_2_Br + 100 µL CsPbI_2_Br + 50 µL RbI, and vortex/stir until homogeneous. After the spinning process, the substrate was transferred immediately into a vacuum flash jig and pumped down to 120 mTorr for 20 s, then to 1.5 Torr for 15 s before releasing the pressure to ambient. The substrate was subsequently annealed on the hot plate at 120 °C for 15 min.

Once the substrate was cooled down, the propane-1,3-diammonium iodide (PDAI_2_) solution (0.5 and 0.7 mg/ml in mixture of 2-propanol and chlorobenzene) was spun on at 4500 rpm with a ramp of 4500 rpm/s for 25 s [S5], followed by annealing on the hot plate at 100 °C for 5 min. The C60 electron transport layer (≈ 16 nm) was deposited via thermal evaporation at 0.1 Å/s. A thin layer for SnO_x_ (≈ 13 nm) was deposited by atomic layer deposition at 83 °C (Beneq TFS200). The hot source vessel (55 °C) is charged with N_2_ for 200 ms, then the pulse of the tetrakis(dimethylamino)tin(IV) (TDMASn) is 100 ms, followed by 12 secs purge with N_2_ at 300 sccm, water pulse for 200 ms and 12 secs purge with N_2_ at 300 sccm [S6]. Followed by Ag or Au thermal evaporation at 0.1 Å/s for the first 10 nm and 1 Å/s for the rest 90 nm. The device has an active area of 0.102 cm². Finally, 112 nm MgF_2_ was thermally evaporated as an antireflection layer with a deposition rate of 3 Å/s. The thicknesses of the C60, SnO_x_ and MgF_2_ layers were calibrated using spectroscopic ellipsometry or cross-sectional scanning electron microscope (SEM). During each experiment, the evaporation rate and layer thickness were monitored with quartz crystal microbalance sensors.

For the certification process, FTO was used as the substrate. The concentration of PDAI_2_ solution has been adjusted to 0.7 mg/ml. 200 nm of Au was used as the electrode (0.1 Å/s for the first 10 nm and 1.5 Å/s for the rest 190 nm). A black shadow mask was used to define the effective working area of the PSCs to 0.06584 cm^2^.

**Semitransparent perovskite solar cells:**

The fabrication process for the semi-transparent perovskite solar cells was similar to that of the opaque perovskite solar cells (PSCs), with a few adjustments. Indium zinc oxide (IZO) contacts were sputtered onto the SnO_x_ layer using a 3-inch IZO ceramic target through a shadow mask, with a radiofrequency power of 30 W, in an Angstrom sputtering system for 60 minutes. An Ag finger, 300 nm thick, was thermally evaporated using a high-precision shadow mask, with a deposition rate of 0.1 Å/s for the first 10 nm and 1.5 Å/s for the remaining 290 nm. The device has an active area of 0.35 cm². Finally, a 112 nm MgF_2_ layer was thermally evaporated as an antireflection coating, with a deposition rate of 3 Å/s.

## S1.2 Characterization

**1.2.1 Kelvin Probe Force Microscopy (KPFM) mapping**

(KP Technology Φ4 ultra-high vacuum Kelvin probe system) was performed over (2 mm x 0.8 mm, 256 data points) under vacuum condition at ambient condition. The tip work function was calibrated on evaporated Au (work function Φ_Au_≈5.4 eV) as Φ_Probe_ = Φ_Au_ + e·V_Probe_. Where Φ_Probe_ represents the probe’s WF, e represents the elementary charge, and V_Probe_ represents the compensation bias. And the sample WF was then obtained as Φ_Sample_ = Φ_Probe_ + e·V_Sample._ Where Φ_Sample_ represents the sample’s WF, and V_Sample_ represents the compensation bias on sample. For the determination of the work function and valence band maximum (VBM), photoemission yield spectroscopy (PYS) was performed. In this method, the square root of the photoemission yield (for metals) or the cube root of the yield (for semiconductors) is plotted as a function of excitation wavelength. A linear fit to the onset region of the spectrum is extrapolated to the x-axis, and the intercept corresponds to the work function (metals) or the VBM position (semiconductors). Importantly, the photoemission measurements were conducted in the same setup as the Kelvin probe measurements, ensuring consistent calibration of the vacuum energy level across both techniques [S7]. Samples preparation: Different ratios of 2PACz and Me-4PACz were spin-coated on the clean ITO substrate. The bottom surface characterization was conducted by tearing off the perovskite film grown on the ITO substrate, following the reported protocol [S8].

**1.2.2 Atomic-force microscopy (AFM)**

The AFM images were detected by Nanosurf CoreAFM. The samples were scanned at the rate of 2s/line with a resolution of 256 points/line at ambient condition. Samples preparation: Different ratios of 2PACz and Me-4PACz were spin-coated on the clean ITO substrate.

**1.2.3 Surface and cross-sectional SEM images**

The SEM images were captured using a FEI Verios SEM system. The images were acquired at a high voltage (HV) of 2.0 to 6.0 kV, a beam current of 86 pA, and magnifications of 10,000x to 60,000x. Samples preparation: (Top surface) perovskite films were spin-coated on different ratios of 2PACz and Me-4PACz spin-coated on the clean ITO substrate; (cross-sectional) PSCs with different ratios of 2PACz and Me-4PACz were prepared.

**1.2.4 Steady-state electroluminescence (EL) and photoluminescence (PL) images**

Steady state PL and EL images were acquired using a hyperspectral imaging tool (Photonetc) equipped with an sCMOS-Si camera. The EL images were captured with devices biased at 1.23 V(~Voc) using Keithely 2400 SMU unit. The laser excitation wavelength was 532 nm, and the illumination intensity was calibrated by matching the short-circuit current to 1-sun under a solar simulator using perovskite solar cells. The PL peak tracking was performed using a Horiba LabRAM system equipped with confocal optics, a silicon charge-couple-device (CCD) detector. The investigated samples were excited with a continuous-wave diode-pumped solid-state (DPSS) 532-nm laser diode. The laser light was focused into the sample surface using a 10 × objective lens with a numerical aperture of 0.25. The PL spectra were recorded every 10 s for 5 min. The laser light intensty was equivalent to ≈ 1.7-sun. The ilumination was conducted from the glass side. Samples preparation: PSCs with different ratios of 2PACz and Me-4PACz were prepared.

**1.2.5 Fourier transform infrared spectroscopy (FTIR) spectra**

FTIR spectra were recorded in the range 400 to 4000 cm^-1^ with a resolution of 1 cm^-1^ by a Bruker VERTEX 80v vacuum spectrometer. Samples preparation: Perovskite films were spin coated on different ratios of 2PACz and Me-4PACz (8.5 mmol/L) on clean Si wafer with native oxide on the surface.

**1.2.6 Grazing Incidence X-Ray Diffraction (GIXRD) measurements**

GIXRD measurements were performed using a D2 Phaser X-Ray Diffractometer with a step size of 0.01° and an integration time of 1 second per step, utilizing Cu Kα as the X-ray source. The measurements were conducted from the top side of the perovskite layer. Samples preparation: Perovskite films were spin coated on different ratios of 2PACz and Me-4PACz on clean ITO.

**1.2.7 X-ray Photoelectron Spectroscopy (XPS)**

XPS results were recorded by photoelectron spectrometer (ESCALAB 250Xi, Thermo Fisher Scientific Inc., USA) with Al Kα radiation (hν = 1486.6 eV) to analyze the element information. Pass energy 30 eV, step 0.1 eV, spot 500μm, 5 to 20 number of scans; energies calibrated to the Au and Fermi edge. Samples preparation: Perovskite (≈ 44.2±7.5 nm) thin films were spin coated (0.4 mmol L^-1^ and 6000 rpm) on different ratios of 2PACz and Me-4PACz on clean ITO.

**1.2.8 Transmittance measurements**

Transmittance spectra were carried out using the IMA PhotoEtc system and a UV-VIS-NIR spectrometer (Perkin Elmer-Lambda 950, Sydney, Australia). Samples were illuminated by a Philips 7724 bulb, controlled via an Olympus TH4-200 power supply. The transmitted light signal was captured using a CMOS camera. Prior to measurement, the light source was allowed to stabilize for 5 minutes. A blank reference image was first taken to record 100% light intensity (blank signal), followed by an image of the sample to capture the transmitted light (sample signal). The relative transmittance was then calculated using the formula: T = (sample signal) / (blank signal). Samples preparation: PSCs with different ratios of 2PACz and Me-4PACz were prepared.

**1.2.9 Quasi-fermi level splitting (QFLS) analysis**

Two separate PL imaging systems were employed in this work. They were both equipped with Si charge-coupled device (CCD) array detectors. For perovskite films and solar cells, the light-emitting diode (LED) excitation wavelength was 430 nm, and the intensity was calibrated using a high-quality working perovskite solar cell. During the measurement process, after changing the illumination intensity, the perovskite samples were kept there for 15 seconds to get the steady-state condition before taking the measurements. Samples preparation: PSCs with different ratios of 2PACz and Me-4PACz were prepared.

**1.2.10 Current-voltage (I-V)**

The *J-V* characteristic of the perovskite cells was measured using solar simulator model #WAVELABS SINUS-220 equipped with a potentiostat source AutolabPGSTAT302N. The light intensity was calibrated at one Sun (100 mW/cm^2^, AM1.5G) using the certified FraunhoferCalLab reference cell. The cells were measured in ambient using the same solar simulator and potentiostat with a controlled temperature at 25 °C. The scanning speed is 200 mV/s.

curves for the space charge limited current (SCLC) of devices with the structure ITO/SAMs/perovskite/Poly-TPD/Ag were measured from 0 to 1.2 V, with a scanning rate of 20 mV/s and a voltage step of 5 mV, using a potentiostat source (Autolab PGSTAT302N). Samples preparation: the samples have the structure of Glass/ITO/SAMs/perovskite/ poly (N, N′-bis-4-butylphenyl-N,N′-bisphenyl) benzidine (Poly-TPD)/Ag. Poly-TPD solution (10 mg/ml in chlorobenzene) was spin-coated onto perovskite films at 3000 rpm for 30 s followed by 10 min annealing at 100°C.

**1.2.11 External quantum efficiency (EQE) measurements**

EQE measurement were conducted using a QE-R quantum efficiency system (Enlitech). The devices, measured without encapsulation and in ambient conditions, were tested over a wavelength range of 300 to 900 nm with a scanning frequency of 80 Hz, measured at short-circuit current. Samples preparation: PSCs with different ratios of 2PACz and Me-4PACz were prepared.

**1.2.12 The operational stability**

The stability of the unencapuslated PSCs was measured under LED illumination (100 mW/cm²) with a UV filter glass placed on top of the cells. This was done in a nitrogen atmosphere at a controlled temperature of 25°C. During the measurement, a bias voltage of 1.0 V, close to the initial maximum power point, was applied, and the current density was continuously monitored.

**2.3.5 COMSOL model**

The model was constructed in COMSOL Multiphysics 6.0 to simulate the influence of the WF and HTL/perovskite interface defect density on the device Voc and FF [S9, S10].

**S2 Supplementary Figures**

The perovskite bottom surface exhibited a valence band at ≈ 5.51 eV (control), and 6.07 eV (target) and a work function of ≈ 4.47 eV(control), and 4.95 eV (target) characterised by the KPFM.


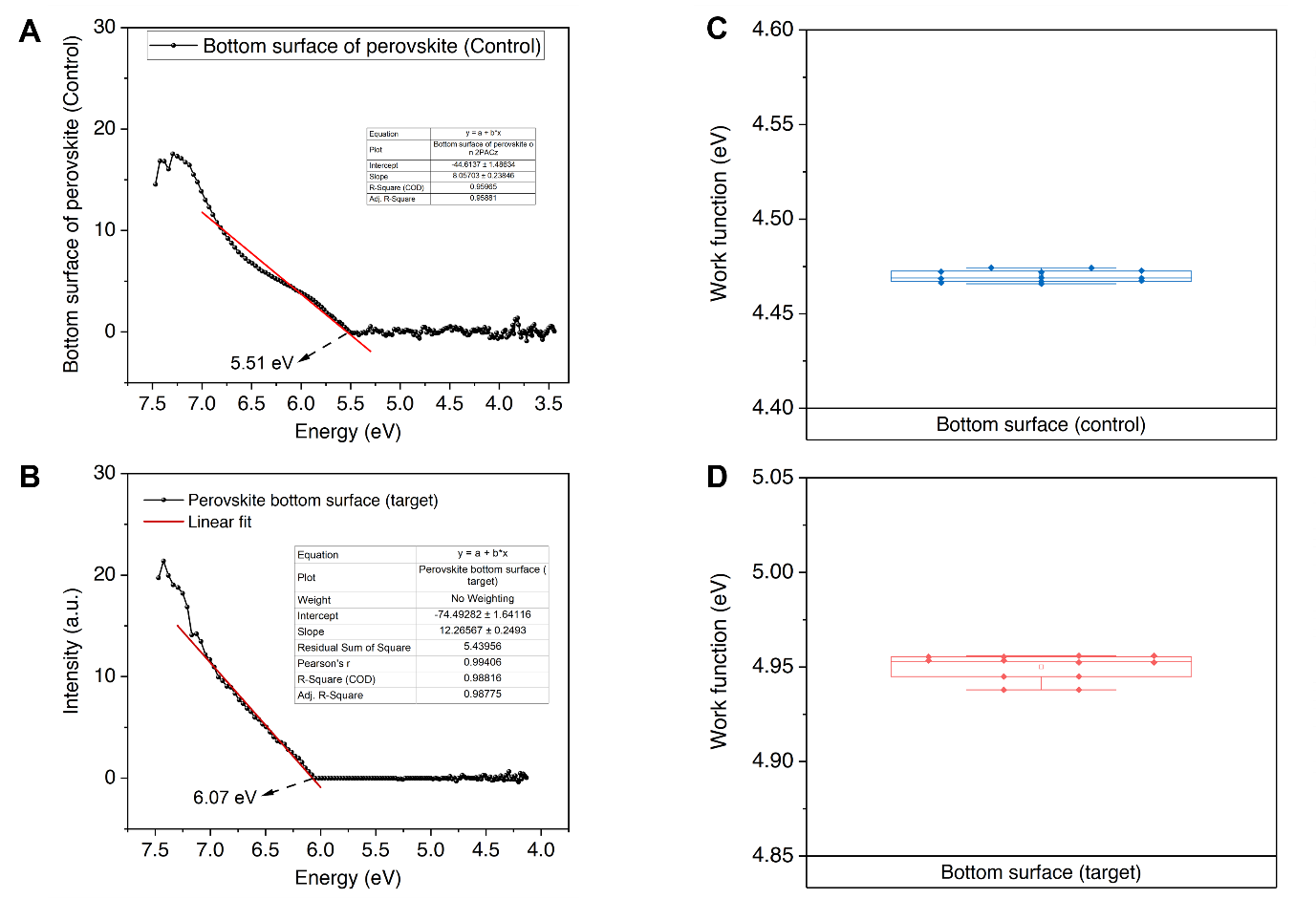


## Fig. S1 The energy level of the perovskite bottom surface. a valence band of the perovskite bottom surface on a 2PACz (control), and b Me-4PACz: 3:1 (target); and the work function of the perovskite bottom surface on c control, d target

3D topographies show that the maximum peak-to-valley height range decreases from ≈ 15 nm on bare ITO to ≈ 12 nm after coating either single-component SAM and is smallest for the mixed SAM (≈ 10 nm), suggesting suppressed vertical aggregation

.

**
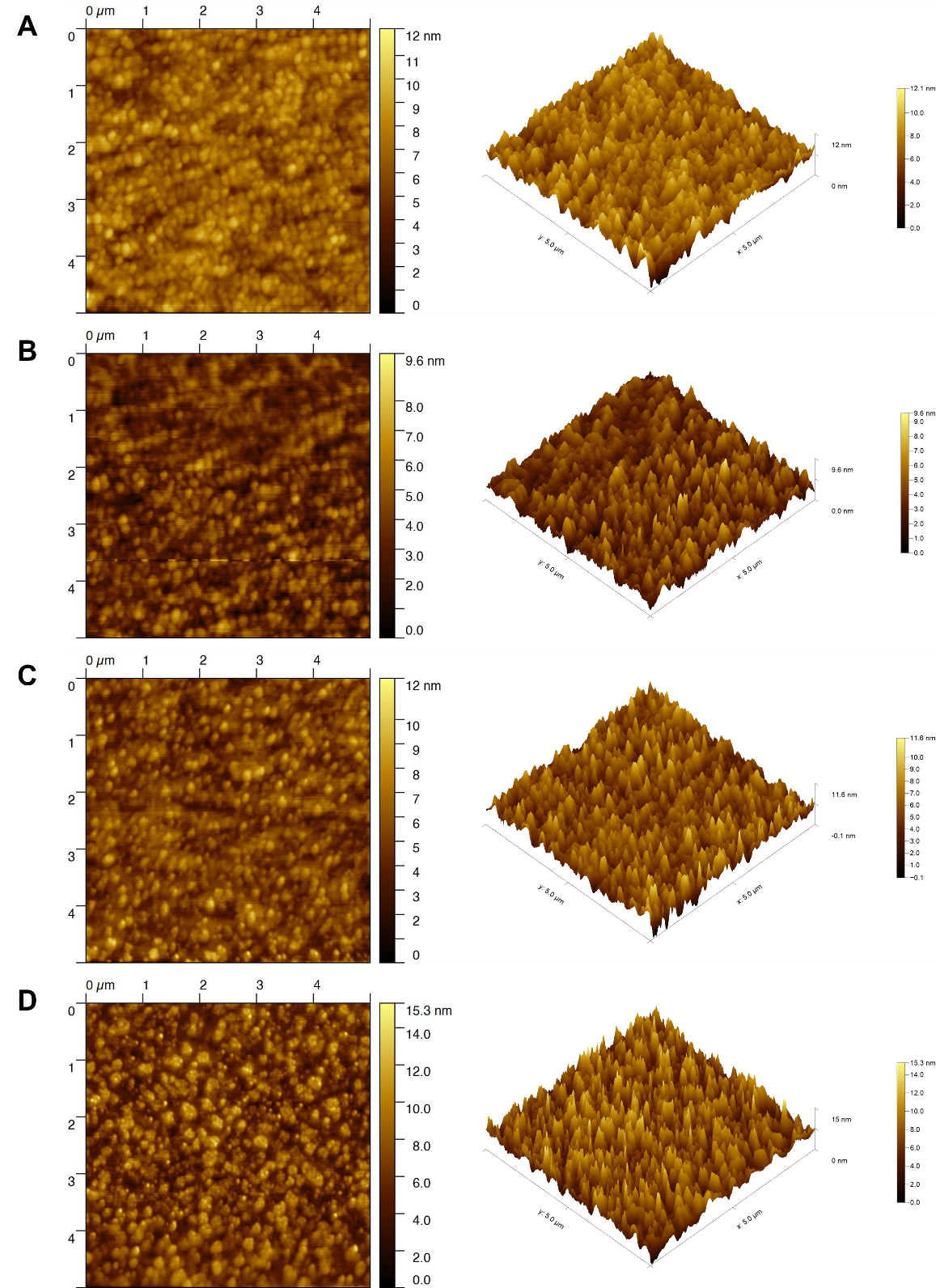
**

## Fig. S2 Atomic force microscopy height image and the three-dimensional surface morphology with and without different ratio of 2PACz: Me-4PACz on indium tin oxide. a 2PACz film; b 2PACz: Me-4PACz (3:1) film; c Me-4PACz film; and d ITO substrate. The detailed information of the surface roughness was presented in the Table S2

The wettability of perovskite on Me-4PACz improved when it was mixed with 2PACz.


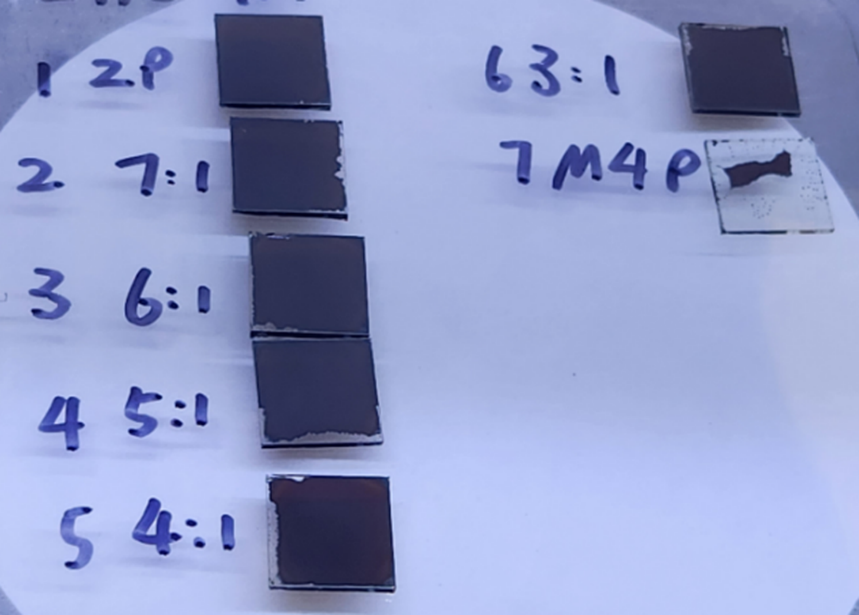


## Fig. S3 The perovskite wettability on different ratios of 2PACz: Me-4PACz mixtures is as follows: Pure 2PACz (top left corner, first), 7:1 (top left corner, second), 6:1 (top left corner, third), 5:1 (top left corner, fourth), 4:1 (top left corner, fifth), 3:1 (top right corner, first), and Pure Me-4PACz (top right corner, second)

Numerous nanovoids were observed on the perovskite top surface when coated on 2PACz.


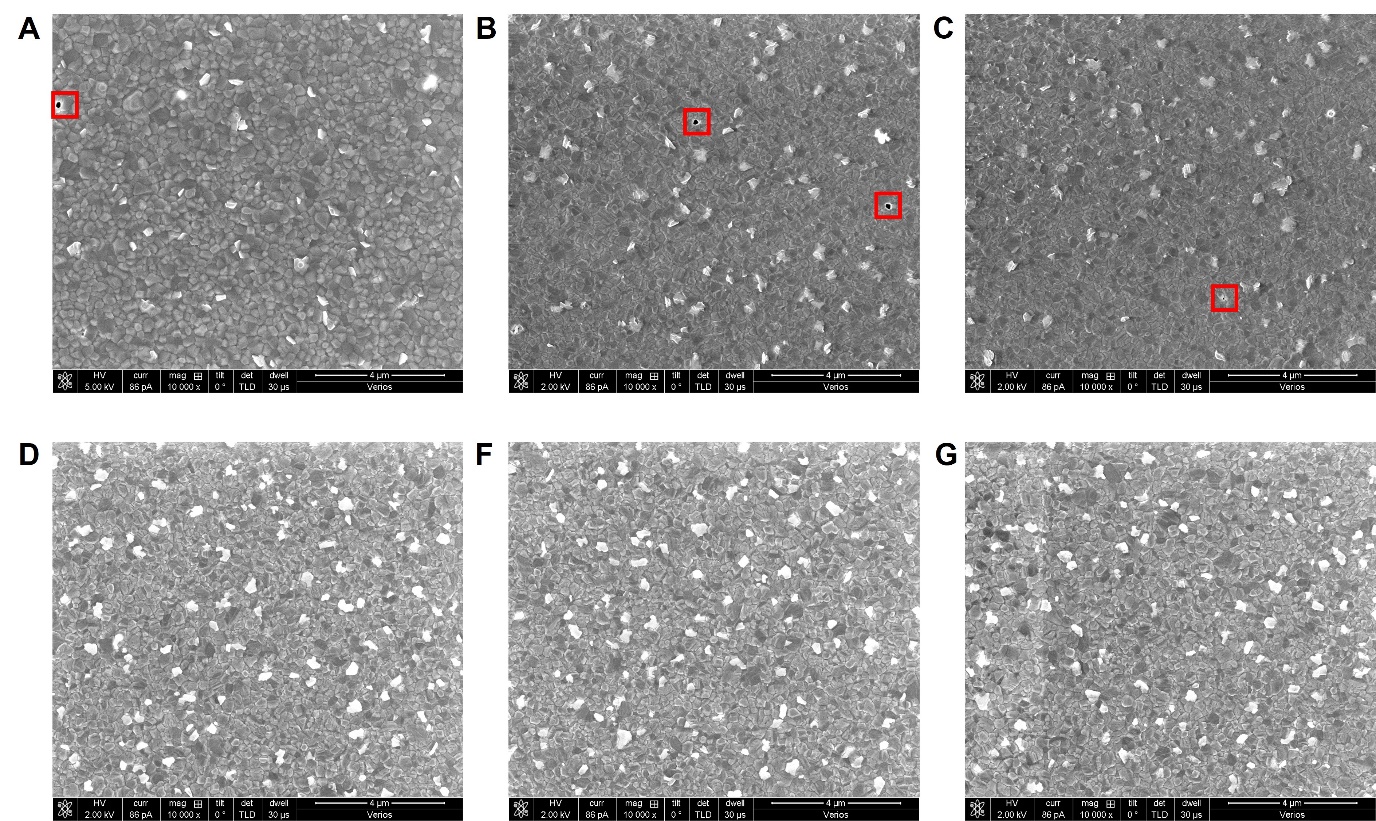


## Fig. S4 SEM images of the perovskite top surface using a-c the 2PACz as hole transport layer (HTL) (The nanovoid are an enclosed in a red square border), and d-g the mixture of 2PACz and Me-4PACz as the HTLs

No significant changes were observed in perovskite grain size according to the SEM images, which averaged around 230 nm in diameter, or in grain boundaries across the different HTLs.


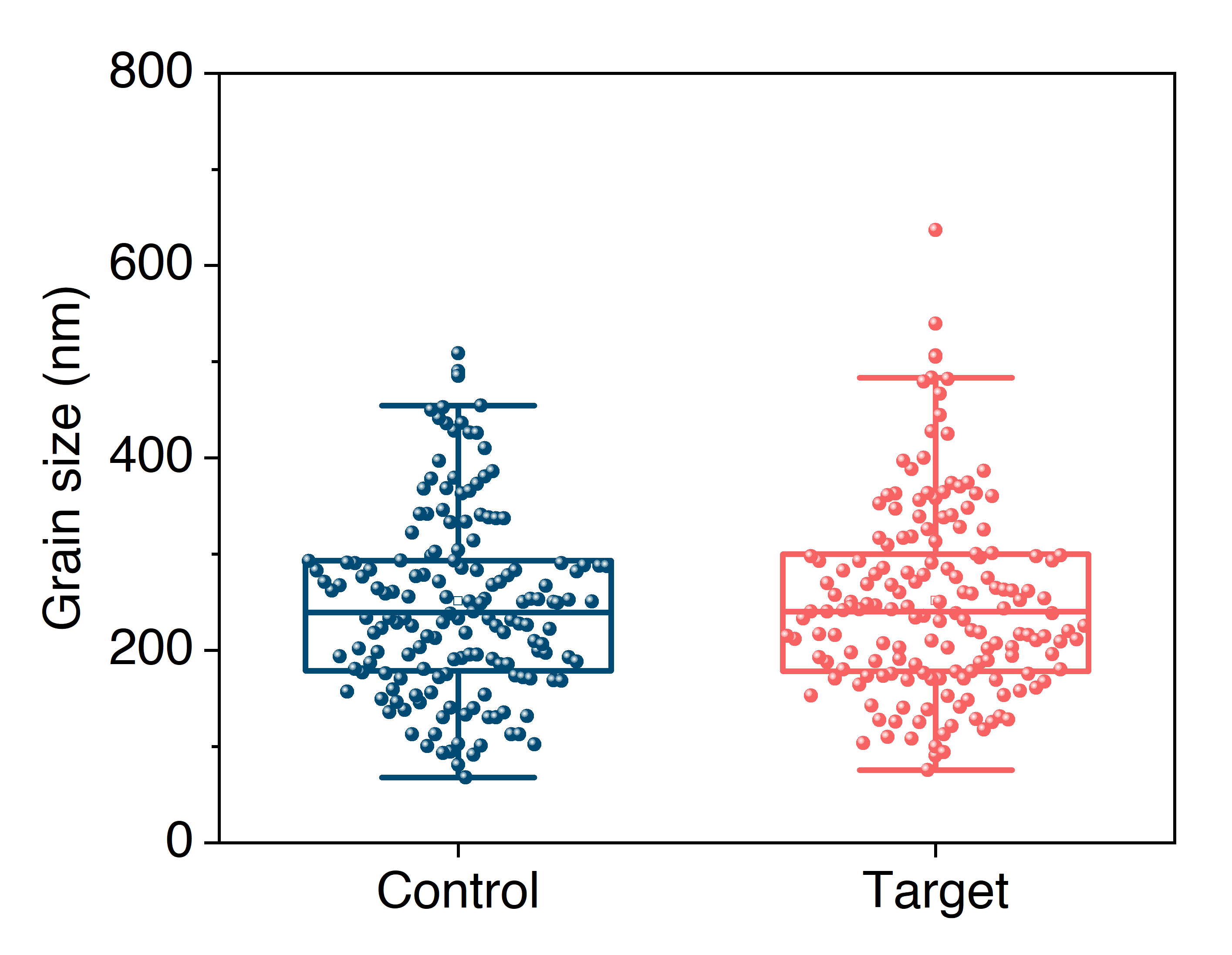


## Fig. S5 Statistics of the perovskite top surface grain sizes using 2PACz (control, blue) and a mixture of 2PACz and Me-4PACz 3:1 (target, red) as HTL, respectively

## The GIXRD results indicate standard perovskite peaks at (001), (011), and (002). We observed a stronger peak at (011) for the perovskite deposited on the mixture of 2PACz and Me-4PACz, suggesting a greater orientation of the crystals in that direction.


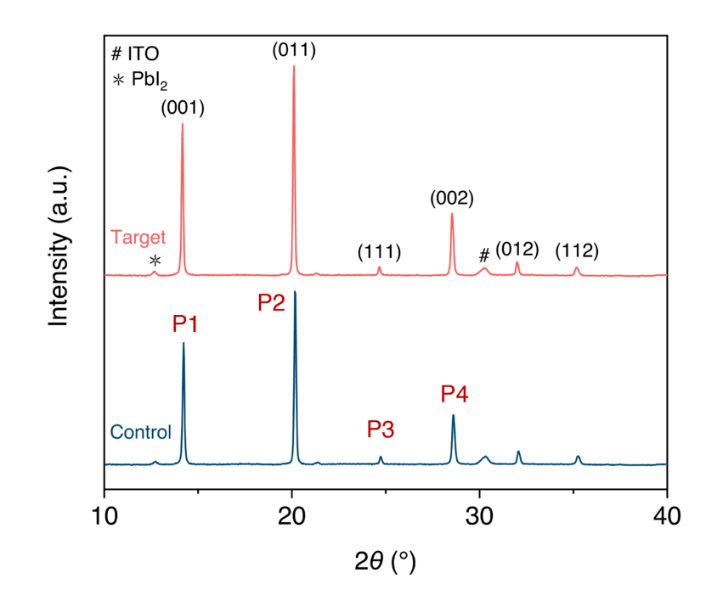


## Fig. S6 The Grazing Incidence X-Ray Diffraction results of the perovskite top surface on different HTLs. 2PACz (control, blue) and a mixture of 2PACz and Me-4PACz 3:1 (target, red). The detailed peak analysis information with the Gaussian fitting was presented in the Table S3.

The FTIR result suggested a shift to a smaller wavenumber as the Me-4pacZ ratio increased.


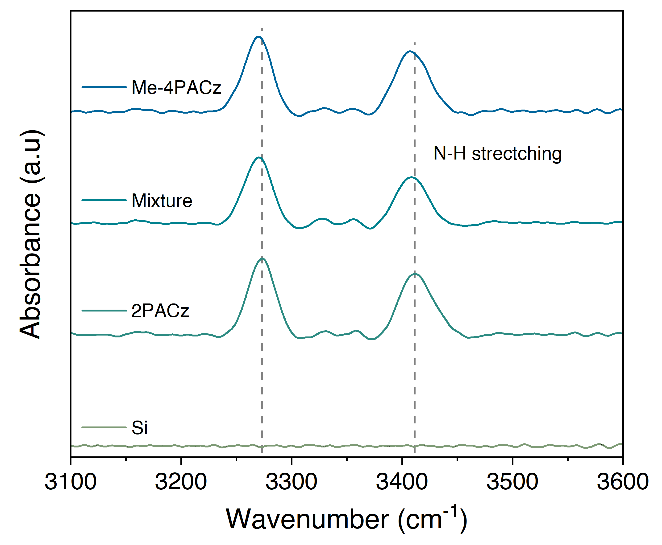


## Fig. S7 FTIR absorbance spectra of the perovskite films coated on different ratio of 2PACz: Me-4PACz deposited on top of Si

## Cross-sectional SEM indicates an average perovskite film thickness of ≈ 44.2±7.5 nm.

##
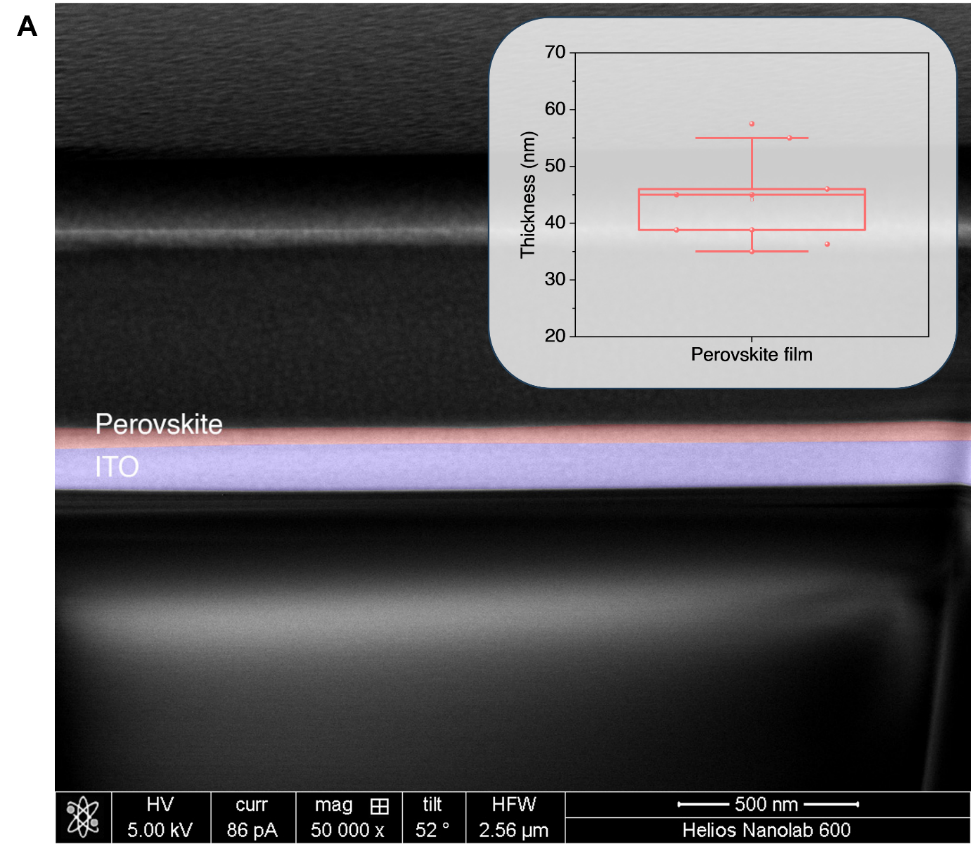


## Fig. S8 Cross-sectional SEM of a perovskite film (reduced perovskite precursor concentration from 1.2 to 0.4 mmol L⁻¹; increased spin-coating rate from 2000 to 6000 rpm) on ITO. Inset: Statistic plot of film thickness at 8 positions (≈ 44.2±7.5 nm).

## Cross-sectional SEM indicates an average perovskite film thickness of ≈ 44.2±7.5 nm.

##
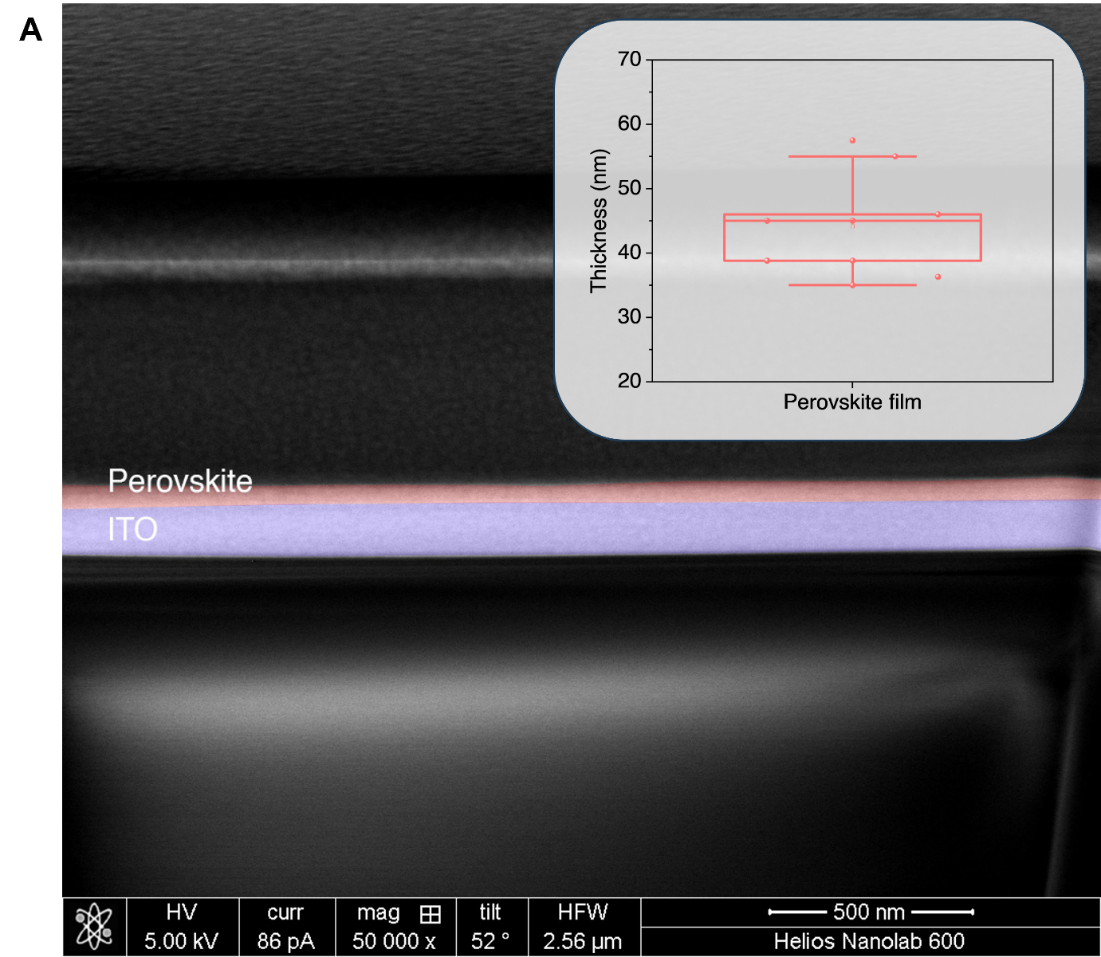


## Fig. S8 Cross-sectional SEM of a perovskite film (reduced perovskite precursor concentration from 1.2 to 0.4 mmol L⁻¹; increased spin-coating rate from 2000 to 6000 rpm) on ITO. Inset: Statistic plot of film thickness at 8 positions (≈ 44.2±7.5 nm)

The X-ray Photoelectron Spectroscopy (XPS) spectra of perovskite films deposited on top of two different HTLs: 2PACz and Me-4PACz. The spectra focus on the Pb 4f peaks, which correspond to the Pb elements in the perovskite material.


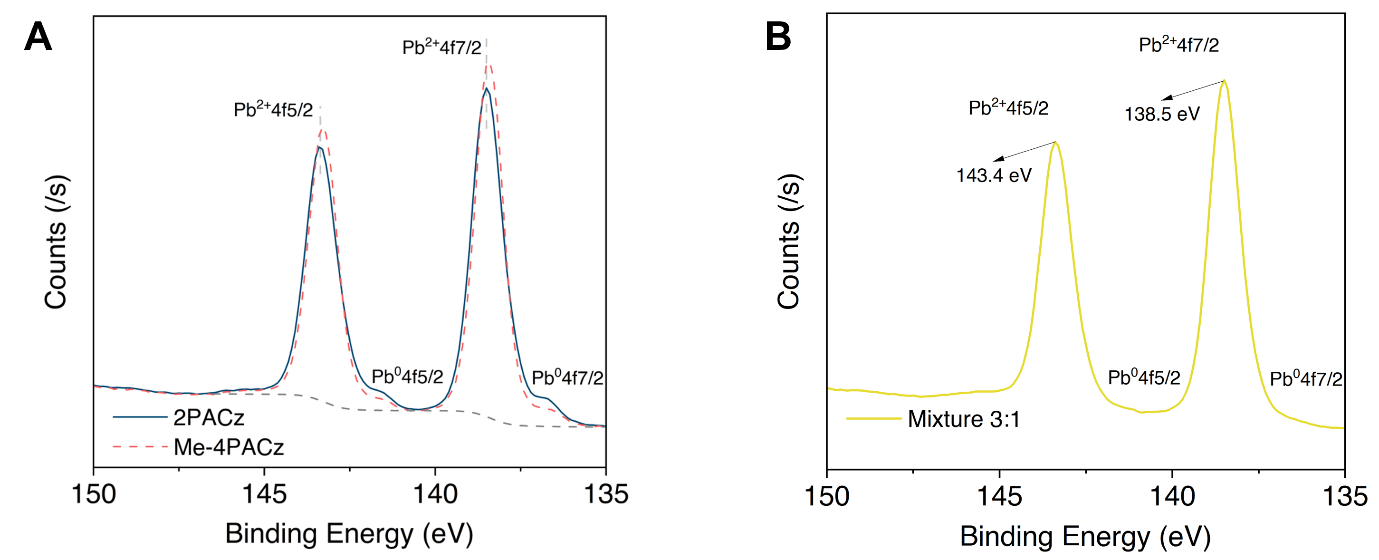


## Fig. S9 XPS spectra of the perovskite films deposited on top of the 2PACz and Me-4PACz as HTL respectively

The trap-state density at the perovskite/HTL interface was estimated using space charge limited current (SCLC) measurement, employing an HTL-only structure of ITO/SAMs/perovskite/ poly (N, N'-bis-4-butylphenyl-N, N'-bisphenyl) benzidine (Poly-TPD)/Ag. The *V_TFL_* values for devices using 2PACz and a mixture of 2PACz: Me-4PACz were determined to be 0.8 V and 0.69 V, respectively.

**
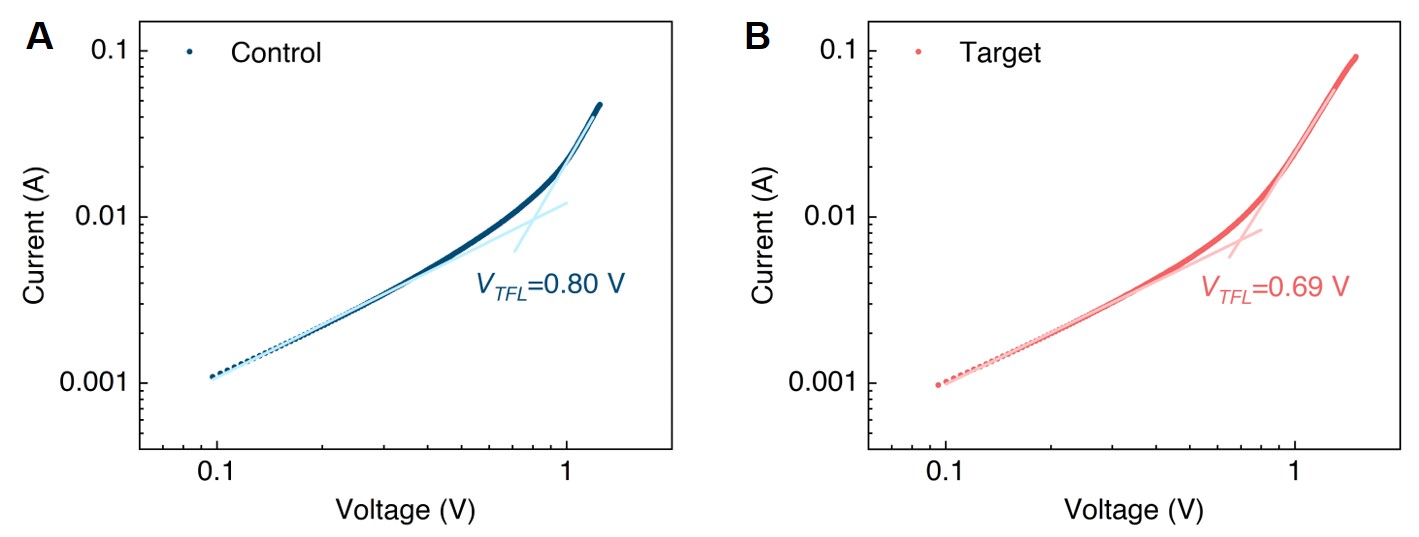
**

## Fig. S10 Dark current-voltage curves of the HTL-only devices using different HTLs. a 2PACz and b the 2PACz: Me-4PACz 3:1 mixture as HTL (ITO/self-assembled monolayers (SAM)s/perovskite/Poly-TPD/Ag)

The thickness of the perovskite films deposited on 2PACz: Me-4PACz mixture was measured using the cross-sectional scanning electron microscope (CSEM). The thickness of the perovskite layer on 2PACz: Me-4PACz was ≈ 400 nm, C60 was ≈ 16 nm, SnO_x_ was ≈ 13 nm.

**
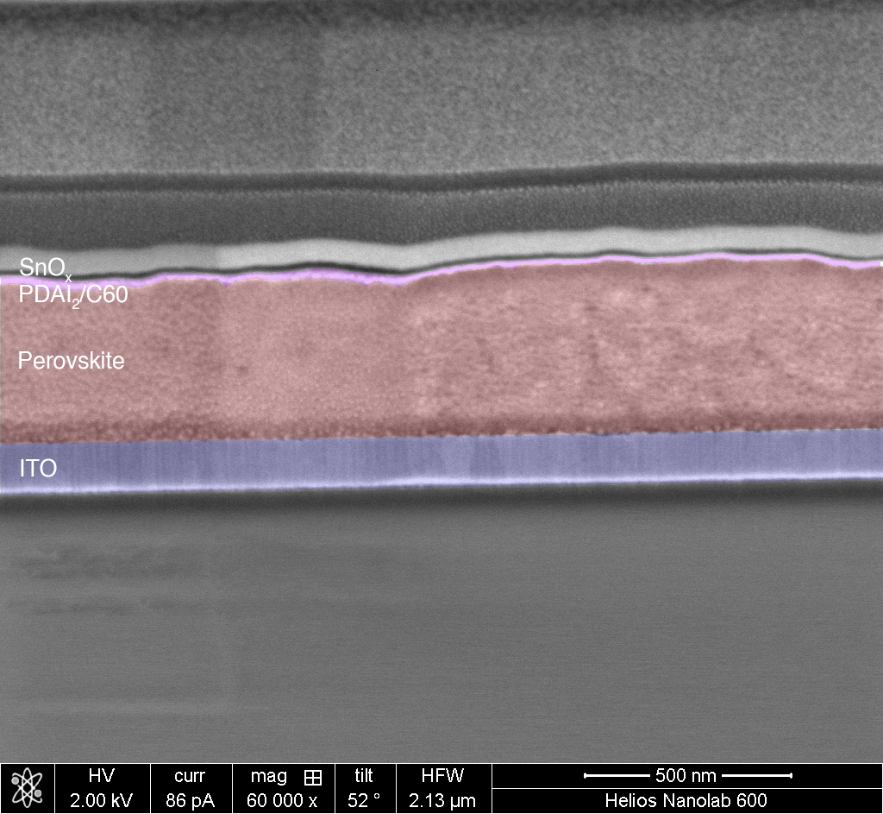
**

## Fig. S11 CSEM images (60,000 x) of perovskite films deposited on 2PACz: Me-4PACz 3:1 mixture

The photovoltaic parameters of different ratio of 2PACz: Me-4PACz based inverted perovskite solar cell (iPSCs), ranging from (1:0 to 3:1).


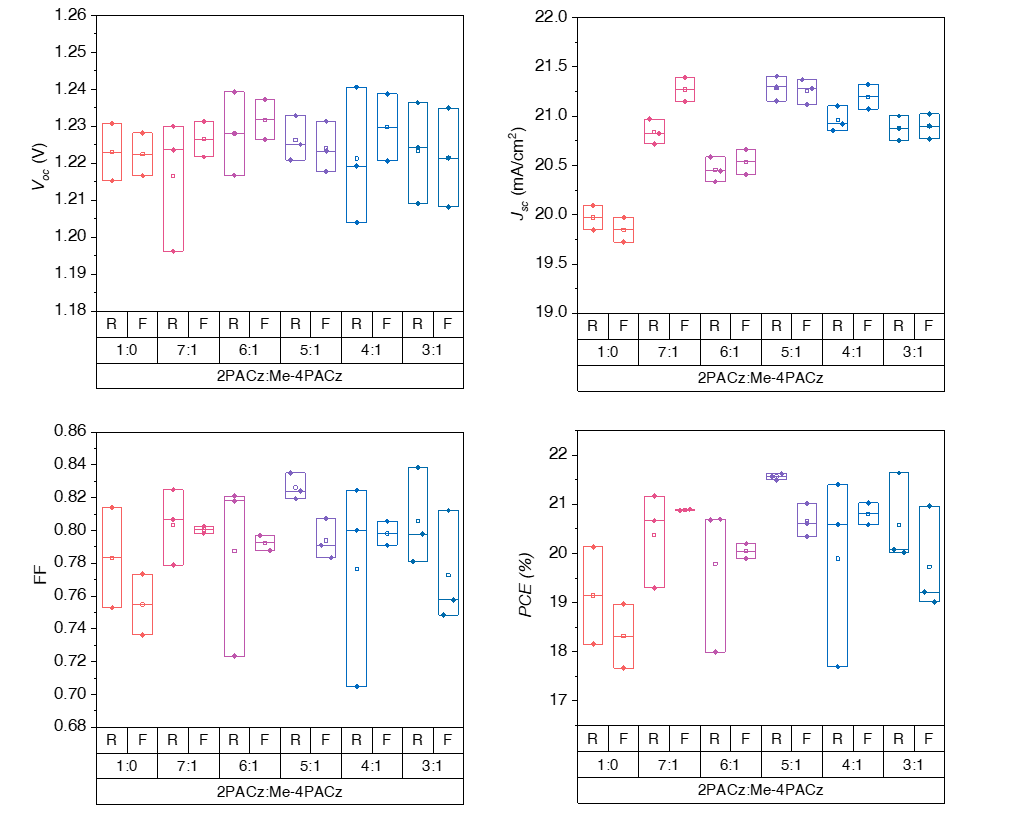


## Fig. S12 Comparison of *J-V* curves parameters of the wide-bandgap perovskite solar cell based on the blended SAM HTL with different ratios ranging from 1:0 to 3:1 (2PACz: Me-4PACz)

The reported photovoltaic performance in this work ranks among the best perovskite solar cells (PSCs) with a bandgap of 1.68 eV. The fill factor (FF) has a 4.15% deficit compared to the Shockley-Queisser limit.

**
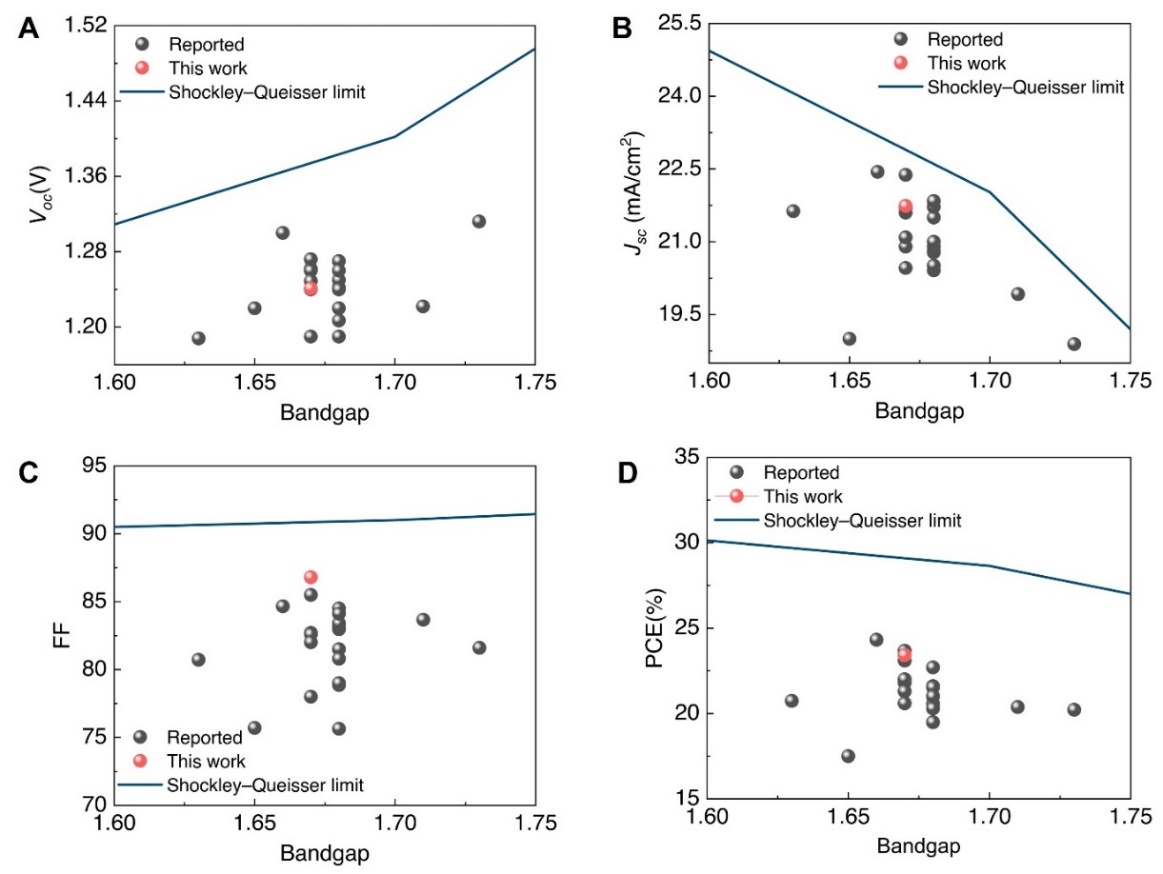
**

**Fig. S13** The reported photovoltaic performance parameters: a, Open-circuit voltage (*V_oc_*), b, Short-circuit current density (*J_sc_*), c, FF and d power conversion efficiency (PCE) of the perovskite solar cells reported in this work and in comparison with historically reported performance for the wide bandgap iPSCs, benchmarked against the Shockley-Queisser limit [S11]

The modest uplift for the target device and the sharper red edge in the EQE spectra indicate reduced interfacial recombination, as reflected by the smaller Urbach energy (≈ 24.8 meV) compared with the control device (≈ 23.73 meV)


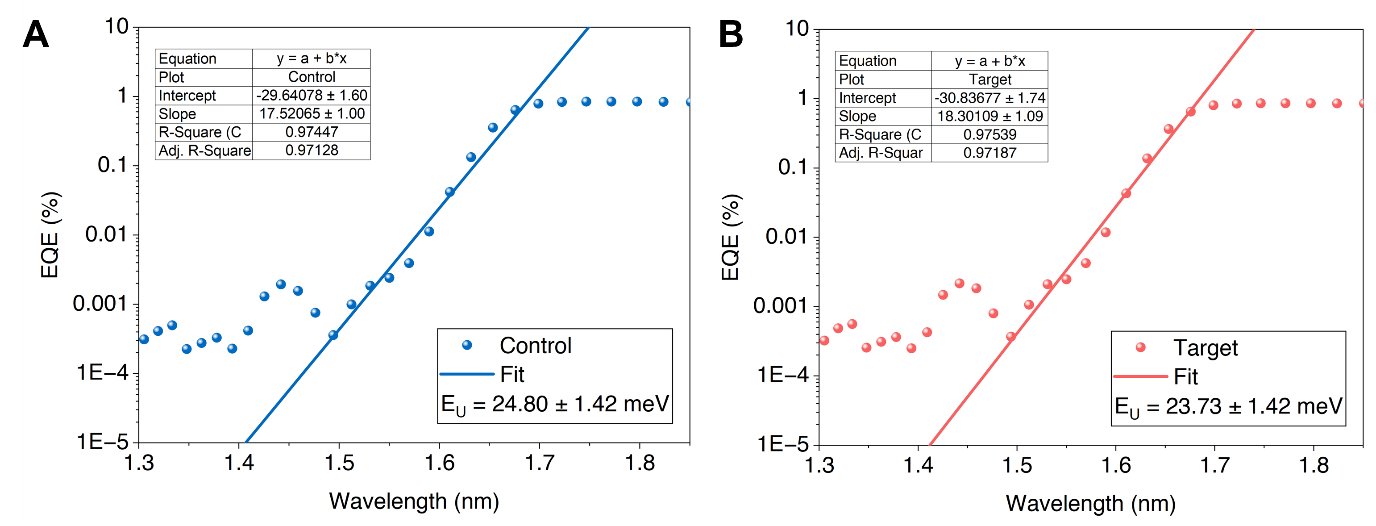


**Fig. S14** EQE spectra and extracted Urbach energies of a 2PACz-based device and b 2PACz: Me-4PACz (3:1)-based device

The cross-sectional SEM indicates a thicker perovskite film ≈ 1143 nm with a improved Jsc but lower *V_oc_* and FF.


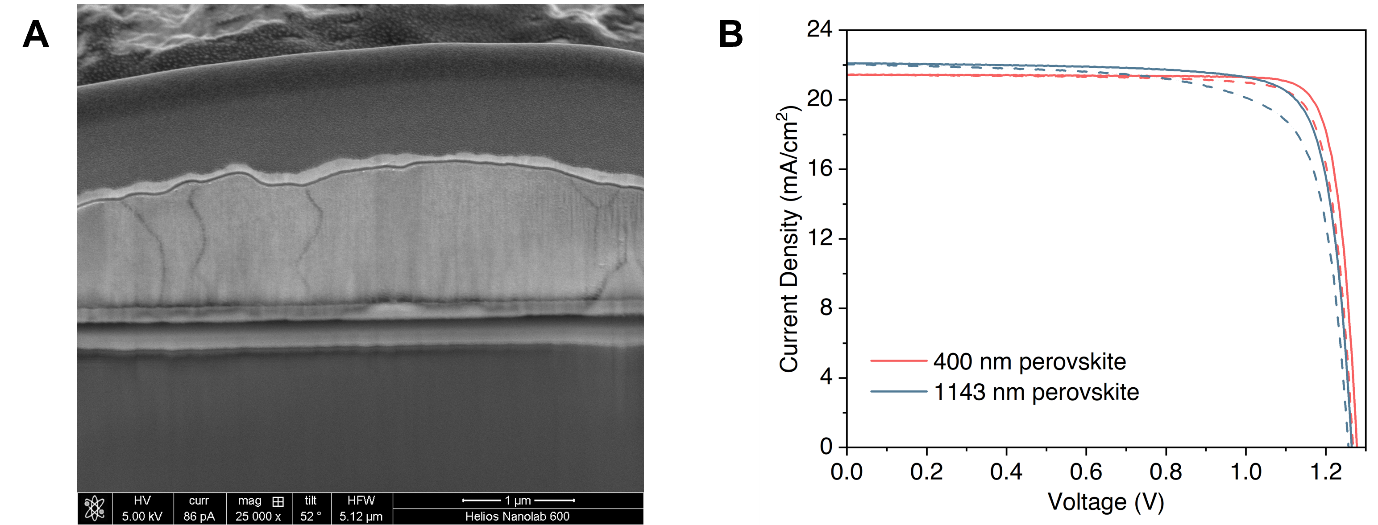


**Fig. S15** a Cross-sectional SEM image of the thicker perovskite solar cell (≈ 1143 nm) together with the b J-V curves comparisons across devices with different perovskite thicknesses. The detailed J-V photovoltaic parameters were presented in the Table S4

The SQ radiative limit for 1.67 eV bandgap is *V_oc_* = 1.37 V and FF = 90.8%. The device measured by 1-sun J-V yields *V_oc_* = 1.252V and FF = 85.6%; Suns-PL (1.259 V) and Suns-Voc (1.251 V) closely match the J-V value, indicating negligible transport penalties. ****

**Fig. S16** Benchmarking *J-V* curve via *J-V*, *Suns- V_oc_*, and Suns-Photoluminescence against the Shockley-Queisser Limit. The detailed J-V photovoltaic parameters were presented in the Table S5

Perovskite film coverage on 2PACz/Me-4PACz is better compared to the Me-4PACz/2PACz.

## Fig. S17 Perovskite coverage on bilayer strategy: 2PACz/Me-4PACz(top), and Me-4PACz/2PACz (bottom)

The 2PACz/Me-4PACz bilayer improved device performance relative to single-layer 2PACz, with the increased FF increasing and decreased hysteresis. Notably, the mixed SAM outperformed the bilayer 2PACz/Me-4PACz, yielding further improvements in *V_oc_*, FF and a pronounced reduction in hysteresis.

## Fig. S18 *J-V* curve comparison of 2PACz, 2PACz: Me-4PACz, and 2PACz/Me-4PACz Bilayer strategy, with the parameters of the best cells. The detailed J-V photovoltaic parameters were presented in the Table S6

The mixed SAM outperformed the pure 2PACz in 1.73 eV perovskite, yielding further improvements in *V_oc_*, *J_sc_*, FF, PCE and a pronounced reduction in hysteresis.

**
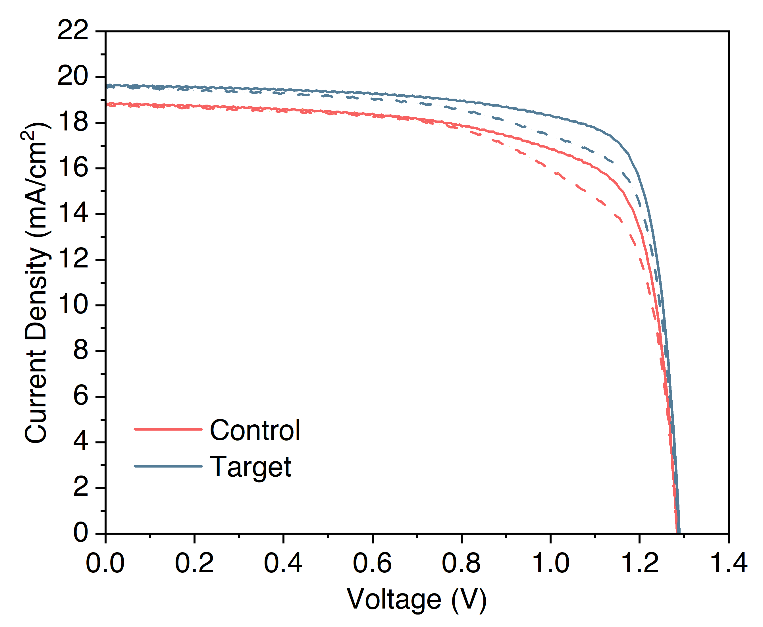
**

## Fig. S19 *J-V* curve comparison of 2PACz, 2PACz: Me-4PACz based 1.73 eV perovskite solar cell. The detailed J-V photovoltaic parameters were presented in the Table S7

To further improve the cell efficiency, we then replaced ITO with Fluorine-doped Tin Oxide (FTO) to enhance the light absorptance. This optimization resulted in improved *J-V* performance, resulting in the cell performance with *V_oc_* of 1.268 V, *J_sc_* of 22.4 mA/cm^2^, FF of 84.7%, and a PCE of 24.07%, with stabilized PCE of 23.47%


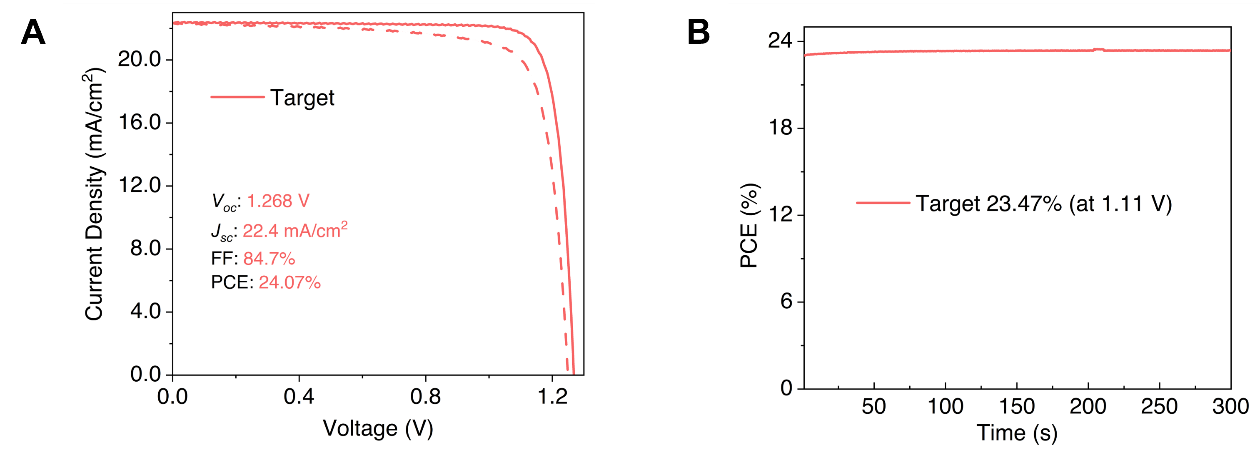


## Fig. S20 a *J-V* curve (solid: reverse scanning; dash line: forward scanning) and b Steady-state efficiency of 2PACz: Me-4PACz mixture as HTL after optimization of PDAI_2_ and changing the bottom substrate to FTO


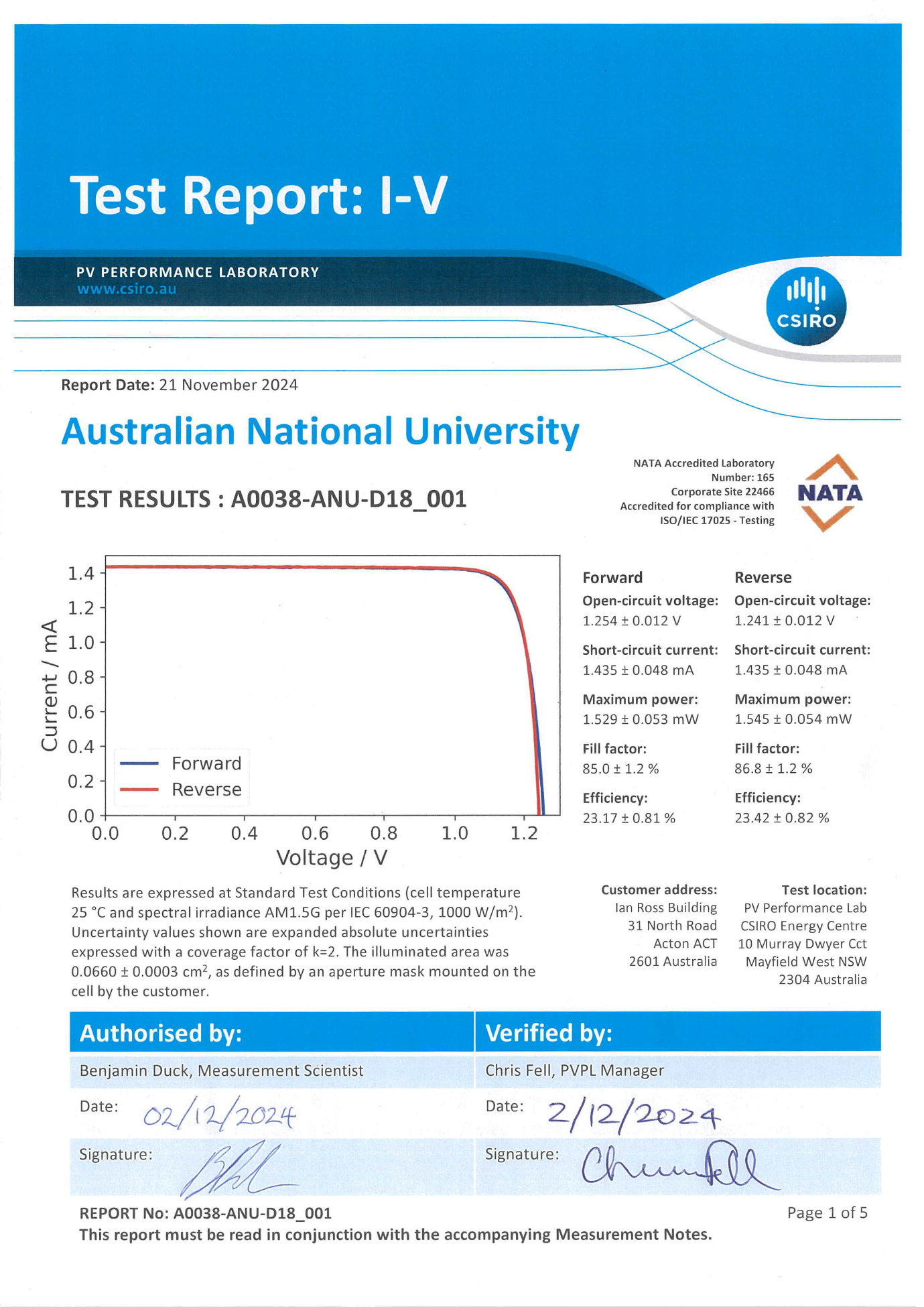


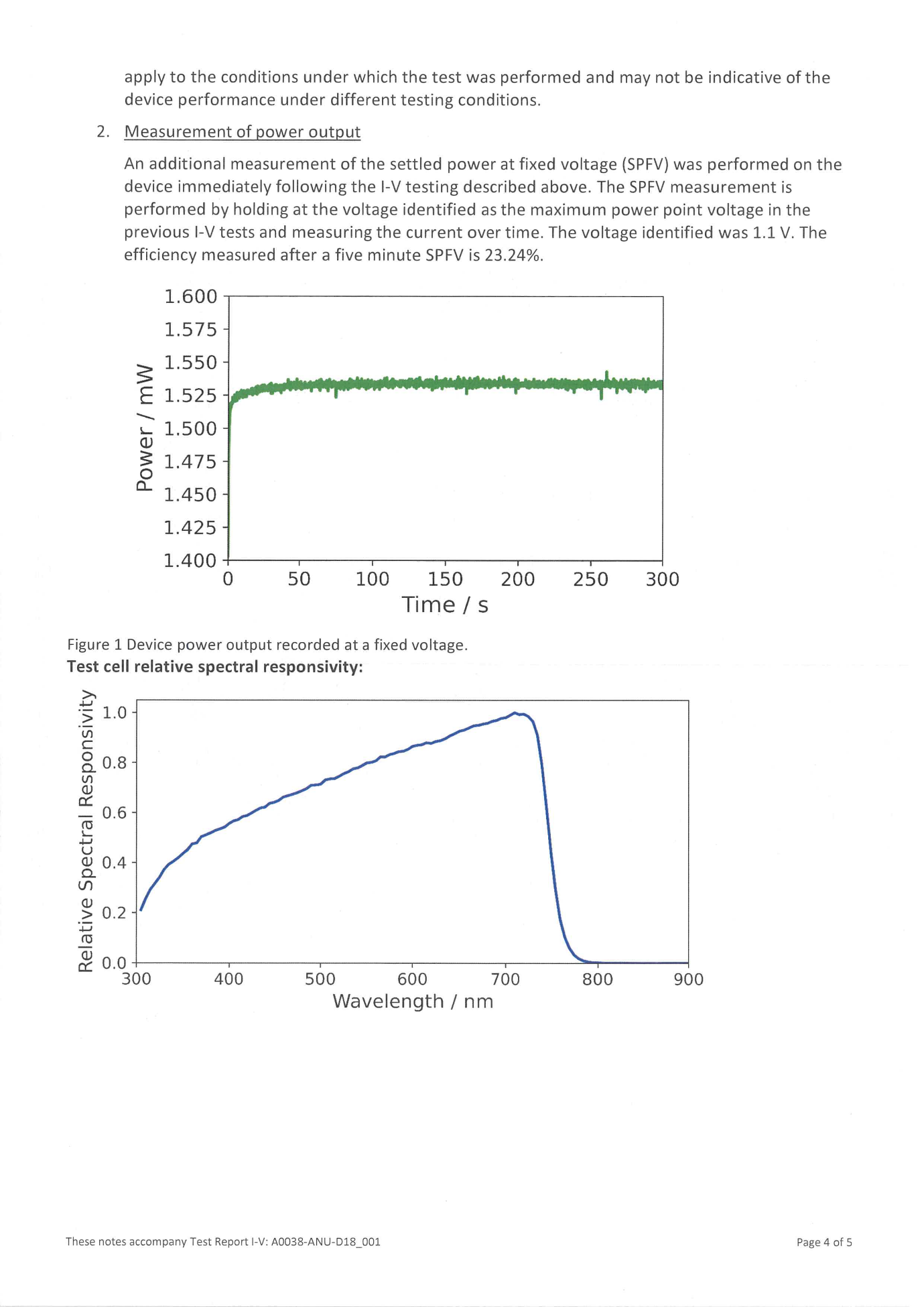


## Fig. S21 Certification report for the photovoltaic performance of the wide bandgap perovskite solar cell reported in this work

The extracted slope value for the control sample was 136 mV/decade which was higher than that of the 2PACz: Me-4PACz HTL device at 120 mV/decade, indicating a higher non-radiative recombination process occurring at the perovskite/HTL interface.

**
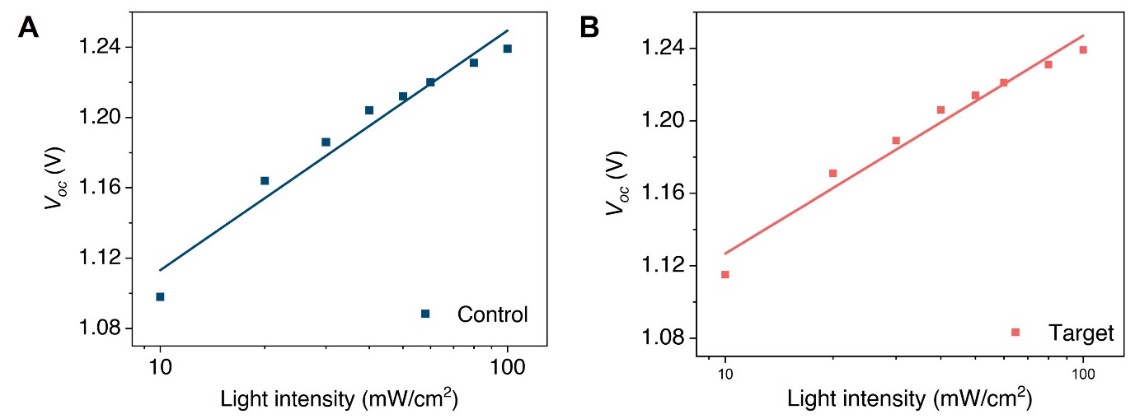
**

**Fig. S22** Light-dependent of *V_oc_* was measured in devices using a 2PACz and b 2PACz: Me-4PACz mixture as HTLs


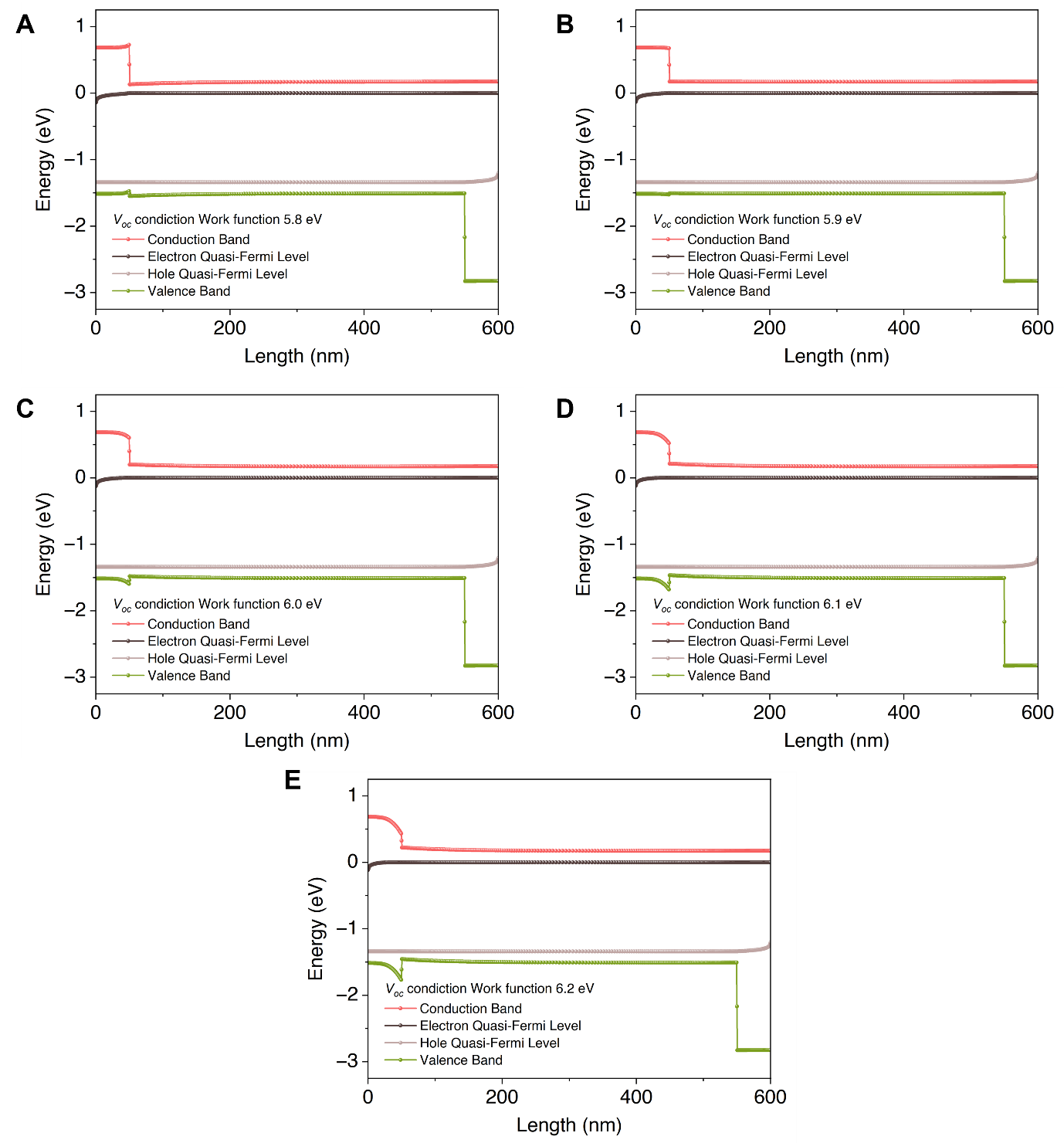


**Fig. S23** Simulated results of the energy band diagram with influence of the work function of the HTL under V_oc_ condition. Panels A–E show band and quasi-Fermi-level profiles across the device at V_oc_​ while the hole-contact work function is increased from 5.8 to 6.2 eV. As the work function rises, the valence band aligns better with the hole contact, reducing the hole-extraction barrier and making the contact more ohmic; at the same time, a positive conduction-band offset (“spike”) appears at the hole side that blocks electrons and suppresses interfacial recombination. The result is a larger and more uniform quasi-Fermi-level splitting throughout the perovskite, implying a higher attainable V_oc_​​. At 5.8 eV the alignment is poor and the quasi-Fermi levels pinch together near the interface, whereas by 6.1-6.2 eV the contact becomes highly selective, and the energy profiles are most favourable

##
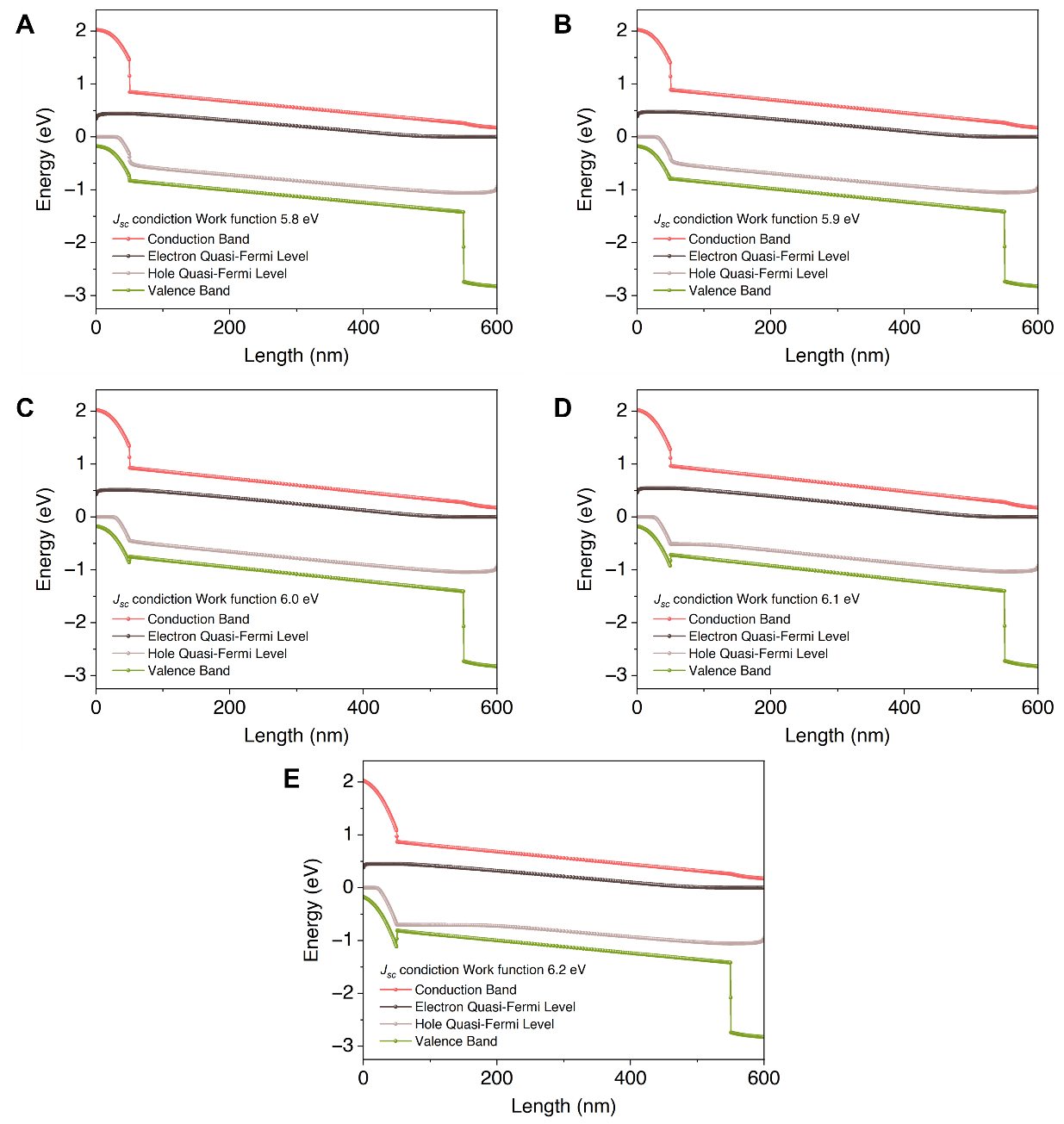


**Fig. S24** Simulated results of the energy band diagram with influence of the work function of the HTL FF under *J_sc_* condition. It shows band and quasi-Fermi-level profiles under *J_sc_* ​ as the hole-contact work function is increased from 5.8 to 6.2 eV. As the work function rises, the perovskite valence band aligns better with the hole contact, removing the hole-extraction barrier (more ohmic) and producing a positive conduction-band “spike” at the hole side that blocks electrons both effects reduce interfacial recombination. This is visible as a larger, more uniform quasi-Fermi-level splitting across the absorber at *J_sc_* ​ ​, which translates to higher usable *J_sc_* ​. At lower work function (5.8 eV) the alignment is poorer and the quasi-Fermi levels pinch together near the hole interface, signalling higher recombination; the 3.7 eV case is worst, creating a “cliff” that impedes hole extraction and would likely cause current loss


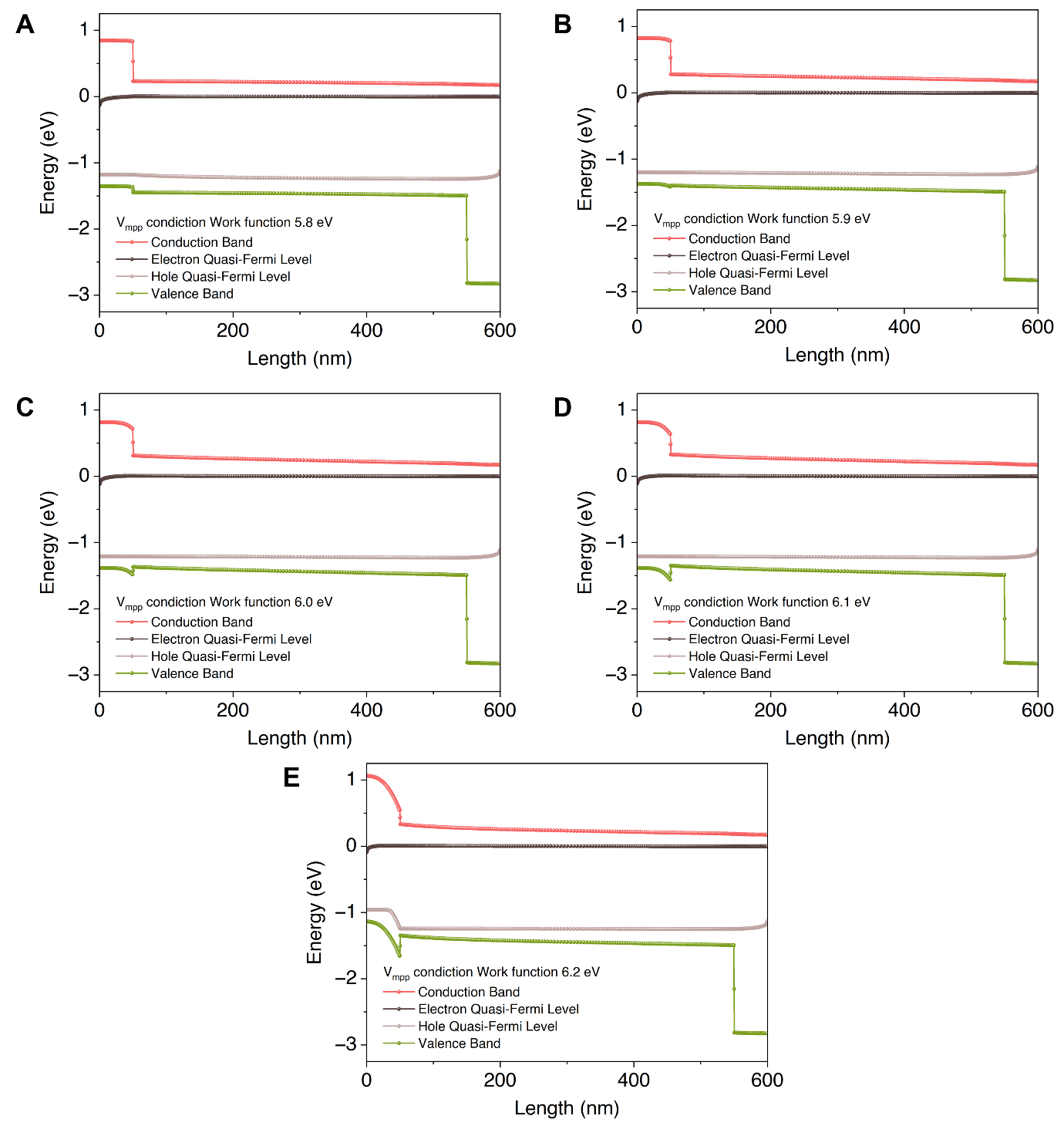


**Fig. S25** Simulated results of the energy band diagram with influence of the work function of the HTL FF under *V_mpp_* condition. These two sets show band and quasi-Fermi-level profiles under *V_mpp_* ​ as the hole-contact work function is increased from 5.8 to 6.2 eV, with one extreme 3.7 eV case at *V_mpp_*. As the work function rises, the perovskite valence band aligns better with the hole contact, removing the hole-extraction barrier (more ohmic) and producing a positive conduction-band “spike” at the hole side that blocks electrons—both effects reduce interfacial recombination. This is visible as a larger, more uniform quasi-Fermi-level splitting across the absorber at both *J_sc_* ​ and *V_mpp_*​, which translates to higher usable *J_sc_* ​, *V_oc_​* ​, and FF. At lower work function (5.8 eV) the alignment is poorer and the quasi-Fermi levels pinch together near the hole interface, signalling higher recombination; the 3.7 eV case is worst, creating a “cliff” that impedes hole extraction and would likely cause S-kinks and current loss. By contrast, ≈6.1–6.2 eV yields the most selective contact good hole collection and strong electron blocking even at *V_mpp_*​​, where forward bias normally relaxes band bending

The blended-SAM devices exhibited only negligible spectral transmittance changes (≈ ±2.5%): increases at 800-980 nm and 1100-1200 nm, and a slight decrease at 980-1100 nm after 430 hours of light stability tracking, implying minimal impact on absorption in the Si bottom cell.


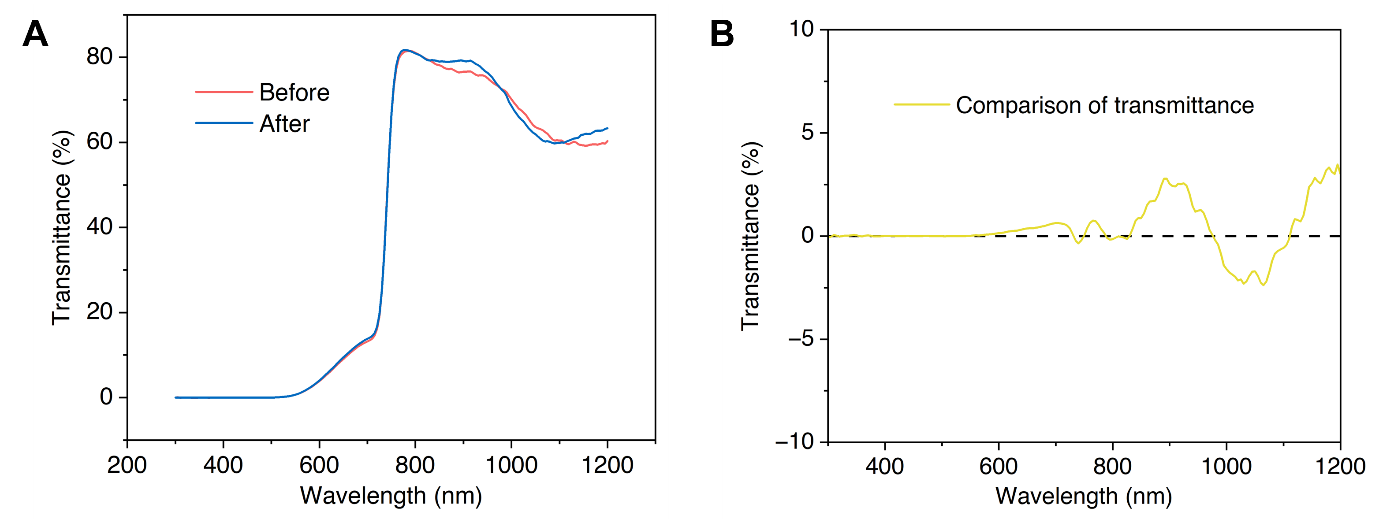


## Fig. S26 Transmittance spectra of the semi-transparent solar cell. a the comparison before and after light stability tracking. b a closer look of the transmission difference

The active area of the Si cell was defined by a 2 x 2 cm^2^ mask.

**
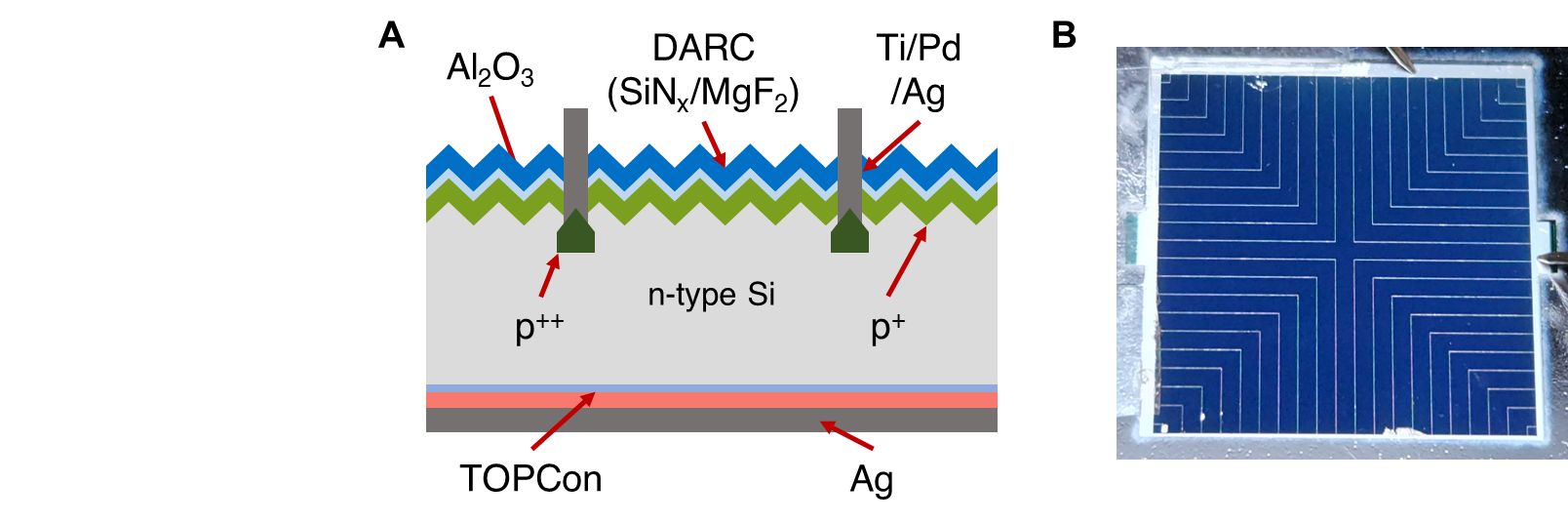
**

## Fig. S27 a Schematic structure of the bottom TOPCon Si solar cell; b Photo of the bottom Si cell design

Parameter evolution indicates that performance losses were dominated by decreases in *J_sc_* and FF.


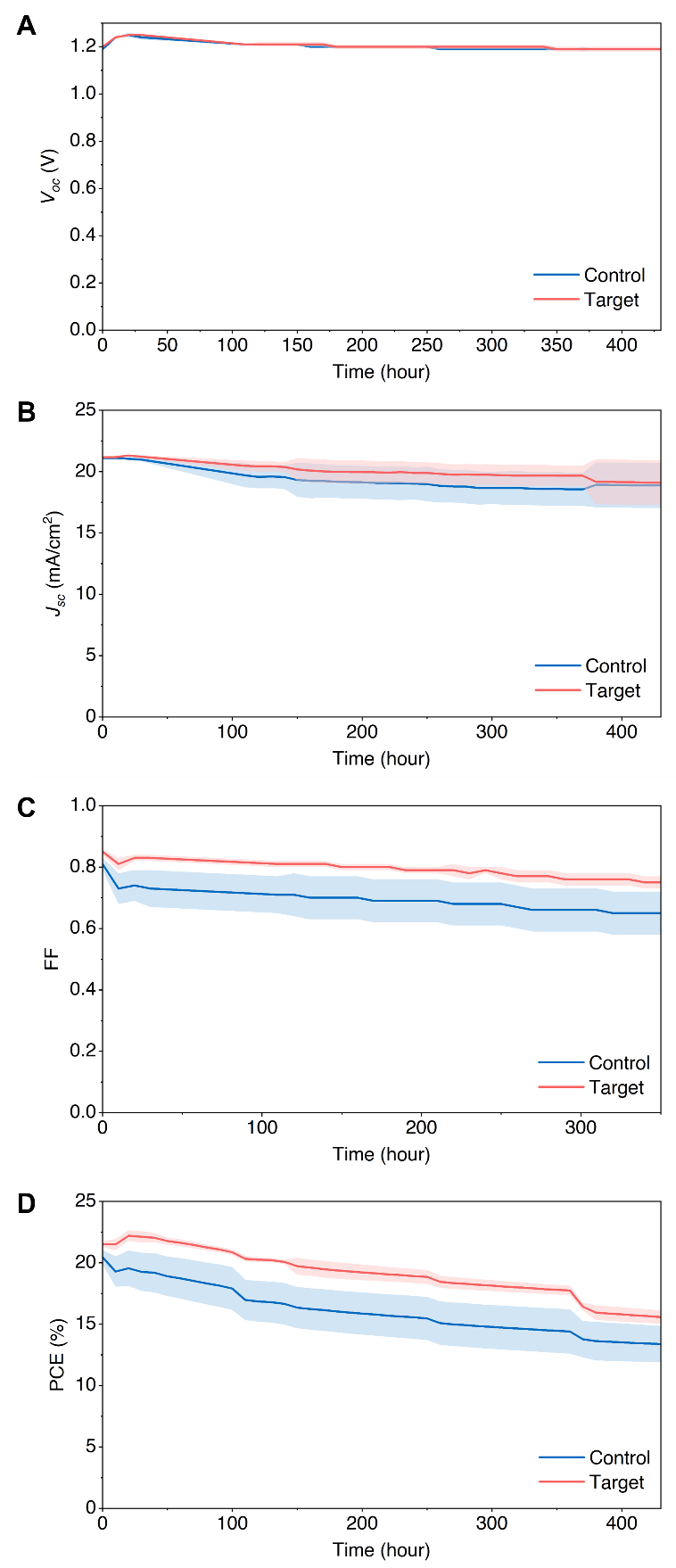


## Fig. S28 The photovoltaic parameters comparison of semi-transparent perovskite solar cells on 2PACz (blue) and 2PACz: Me-4PACz (3:1) (red) after 430 hours of light tracking at 25°C in N_2_ atmosphere. Three cells were measured in each condition

# S3 Supplementary Tables

## Table S1 Efficient wide bandgap inverted perovskite solar cells reported in the past two years

| **Year** | **Bandgap (eV)** | ***V_oc_* (V)** | ***J_sc_* (mA/cm^2^)** | **FF** | **PCE (%)** | **Certification** | **Refs** |
| --- | --- | --- | --- | --- | --- | --- | --- |
| 2023 | 1.71 | 1.222 | 19.92 | 83.67 | 20.38 | No | [S12] |
| 2023 | 1.68 | 1.240 | 20.78 | 75.64 | 19.49 | No | [S13] |
| 2023 | 1.68 | 1.242 | 20.41 | 82.97 | 21.02 | No | [S14] |
| 2023 | 1.68 | 1.250 | 21.50 | 84.50 | 22.70 | No | [S15] |
| 2023 | 1.68 | 1.220 | 21.72 | 81.50 | 21.59 | No | [S16] |
| 2023 | 1.73 | 1.312 | 18.89 | 81.60 | 20.22 | No | [S17] |
| 2023 | 1.67 | 1.190 | 21.60 | 78.00 | 20.60 | No | [S18] |
| 2023 | 1.68 | 1.190 | 21.84 | 83.10 | 21.60 | No | [S19] |
| 2023 | 1.68 | 1.270 | 20.51 | 78.86 | 20.58 | No | [S20] |
| 2023 | 1.65 | 1.220 | 19.00 | 75.70 | 17.50 | No | [S21] |
| 2023 | 1.68 | 1.240 | 20.81 | 83.40 | 21.53 | No | [S22] |
| 2023 | 1.67 | 1.262 | 20.90 | 82.70 | 21.80 | No | [S23] |
| 2023 | 1.68 | 1.207 | 20.90 | 80.80 | 20.30 | No | [S24] |
| 2023 | 1.63 | 1.188 | 21.63 | 80.72 | 20.74 | No | [S25] |
| 2023 | 1.67 | 1.260 | 20.46 | 82.60 | 21.30 | No | [S26] |
| 2023 | 1.67 | 1.272 | 21.09 | 82.01 | 22.00 | No | [S27] |
| 2024 | 1.68 | 1.260 | 21.00 | 79.00 | 21.00 | No | [S28] |
| 2024 | 1.67 | 1.241 | 21.93 | 84.29 | 22.95 | Yes | [S29] |
| 2024 | 1.67 | 1.249 | 22.44 | 84.66 | 23.67 | Yes | [S30] |
| 2024 | 1.66 | 1.290 | 22.20 | 83.91 | 24.07 | Yes | [S31] |
| **2024** | **1.67** | **1.268** | **22.4** | **84.7** | **24.07** | **In house measurement** | **This work** |
| **2024** | **1.67** | **1.241** | **21.74** | **86.80** | **23.42** | **(certified)** |  |

## Table S2 Surface roughness parameters comparison on with and without different ratio of 2PACz: Me-4PACz on indium tin oxide. Where S_a_ stands for arithmetic mean height; S_q_ stands for root mean square roughness; and S_y_ stands for maximum height of the surface

| **Condition** | **Sa(pm)** | **Sq(pm)** | **Sy(nm)** |
| --- | --- | --- | --- |
| 2PACz | 862.4 | 1074.5 | 8.5 |
| 2PACz: Me-4PACz (3:1) | 808.2 | 1010.1 | 7.7 |
| Me-4PACz | 893.4 | 1144.7 | 10.2 |
| ITO | 1471.1 | 1819.3 | 14.3 |

## Table S3 The detailed peak analysis information with the Gaussian fitting of the GIXRD results of the perovskite top surface on different HTLs. 2PACz (control) and a mixture of 2PACz and Me-4PACz 3:1 (target)

| Model | Gaussian (Target) | | | | | |
| --- | --- | --- | --- | --- | --- | --- |
| Equation | y = y_0_ + A/ (w*sqrt (pi/ (4*ln (2)))) * exp (-4*ln (2) *(x-x_c_) ^2/w^2) | | | | | |
| Plot | Peak1(Intensity) | | Peak2(Intensity) | | Peak3(Intensity) | Peak4(Intensity) |
| y_0_ | 127.49 ± 19.51 | | 127.49 ± 19.51 | | 127.49 ± 19.51 | 127.49 ± 19.51 |
| x_c_ | 14.12 ± 4.18 E-4 | | 20.10 ± 3.07 E-4 | | 24.66 ± 0.01 | 28.54 ± 0.001 |
| A | 6929.25 ± 52.90 | | 10275.42 ± 54.47 | | 456.63 ± 58.18 | 4054.24 ± 63.31 |
| w | 0.11 ± 9.86 E-4 | | 0.12 ± 7.25 E-4 | | 0.14 ± 0.02 | 0.16 ± 0.003 |
| Reduced Chi-Sqr | 417326.40 | | | | | |
| R-Square (COD) | 0.99 | | | | | |
| Adj. R-Square | 0.99 | | | | | |
|  | | | | | | |
| Model | Gaussian (Control) | | | | | |
| Equation | y = y_0_ + A/ (w*sqrt (pi/ (4*ln (2)))) * exp (-4*ln (2) *(x-x_c_) ^2/w^2) | | | | | |
| Plot | Peak1(Intensity) | Peak2(Intensity) | | Peak3(Intensity) | | Peak4(Intensity) |
| y_0_ | 132.93 ± 18.57 | 132.93 ± 18.57 | | 132.93 ± 18.57 | | 132.93 ± 18.57 |
| x_c_ | 14.23 ± 5.20 E-4 | 20.17 ± 3.56 E-4 | | 24.73 ± 0.009 | | 28.61 ± 0.001 |
| A | 5986.45 ± 52.41 | 9334.14 ± 53.58 | | 422.90 ± 56.81 | | 3493.75 ± 62.1 |
| w | 0.12 ± 0.001 | 0.13 ± 8.40 E-4 | | 0.14 ± 0.02 | | 0.17 ± 0.003 |
| Reduced Chi-Sqr | 377012.74 | | | | | |
| R-Square (COD) | 0.98 | | | | | |
| Adj. R-Square | 0.98 | | | | | |
|  | | | | | | |

**Table S4** Comparison of *J-V* curves parameters among different inverted perovskite solar cells with different perovskite thicknesses on 2PACz: Me-4PACz 3:1 HTL

| Condition | | *V_oc_* (V) | *J_sc_* (mA/cm^2^) | FF | PCE (%) | Hysteresis (%) |
| --- | --- | --- | --- | --- | --- | --- |
| Perovskite  ≈ 400 nm | Average | 1.27±0.01 | 21.24±0.16 | 85.50±0.41 | 23.14±0.30 | 3.13±0.46 |
|  | Champion | 1.28 | 21.45 | 86.00 | 23.57 | 3.65 |
| Perovskite  ≈ 1143 nm | Average | 1.24±0.01 | 22.04±0.19 | 76.16±3.35 | 20.91±1.25 | 12.18±3.30 |
|  | Champion | 1.26 | 22.10 | 80.80 | 22.58 | 8.24 |

## Table S5 Benchmarking *V_oc_* and FF via *J-V*, *Suns- V_oc_*, and Suns-Photoluminescence against the Shockley-Queisser Limit

|  | ***V_oc_*** | **FF** |
| --- | --- | --- |
| SQ limit | 1.370 | 90.8 |
| JV | 1.252 | 85.6 |
| Suns-*V_oc_* | 1.251 | 87.1 |
| Suns-PL | 1.259 | 86.6 |

## Table S6 Comparison of *J-V* curves parameters among different inverted perovskite solar cells using 2PACz, 2PACz: Me-4PACz, or 2PACz/Me-4PACz as the hole transport layer

| Condition | | *V_oc_* (V) | *J_sc_* (mA/cm^2^) | FF | PCE (%) | Hysteresis (%) |
| --- | --- | --- | --- | --- | --- | --- |
| 2PACz | Average | 1.26±0.01 | 21.23±0.02 | 80.49±5.10 | 21.67±1.42 | 5.50±3.26 |
|  | Champion | 1.27 | 21.26 | 84.6 | 22.67 | 6.15 |
| 2PACz: Me-4PACz 3:1 | Average | 1.27±0.01 | 21.35±0.09 | 85.50±0.41 | 23.26±0.27 | 3.13±0.46 |
|  | Champion | 1.28 | 21.45 | 86.00 | 23.57 | 3.65 |
| 2PACz/Me-4PACz | Average | 1.27±0.01 | 21.37±0.25 | 84.54±0.75 | 22.90±0.43 | 12.18±3.30 |
|  | Champion | 1.27 | 21.16 | 85.46 | 22.96 | 6.46 |

## Table S7 Comparison of *J-V* curves parameters among 2APCz and 2PACz: Me-4PACz (3:1) mixture of the 1.73 eV perovskite solar cells

| Condition | | *V_oc_* (V) | *J_sc_* (mA/cm^2^) | FF | PCE (%) | Hysteresis (%) |
| --- | --- | --- | --- | --- | --- | --- |
|  | Average | 1.28±0.01 | 18.90±0.07 | 72.00±0.80 | 17.48±0.18 | 9.12±0.38 |
|  | Champion | 1.28 | 18.85 | 73.11 | 17.70 | 8.43 |
| 2PACz: Me-4PACz 3:1 | Average | 1.29±0.02 | 19.17±0.12 | 78.61±1.20 | 19.47±0.24 | 5.85±1.05 |
|  | Champion | 1.26 | 19.12 | 80.26 | 19.85 | 4.95 |

## Table S8 Fitting parameters of the light-dependent of *V_oc_*

|  | Slope (V/decade) | Residual sun of squares |
| --- | --- | --- |
| Control | 0.136 | 6.14E-4 |
| Target | 0.120 | 3.67E-4 |

## Table S9 COMSOL Simulation parameters [S10]

| **Parameter** | **Value** |
| --- | --- |
| Relative permittivity | 64 |
| Thickness | 500 nm |
| Effective density of states for conduction band | 5 x 10^18^ cm^-3^ |
| Effective density of states for valence band | 5 x 10^18^ cm^-3^ |
| Electron affinity | 3.9 eV |
| Bandgap | 1.67 eV |
| Electron mobility | 1 cm^2^/(V·s) |
| Hole mobility | 0.01 cm^2^/(V·s) |
| Dopant density | 1.4 x 10^12^ cm^-3^ |

| **Parameter** | **Electron transport layer** | **Hole transport layer** |
| --- | --- | --- |
| Relative permittivity | 24 | 3 |
| Bandgap | 3 eV | 2.4 eV |
| Effective density of states for conduction band | 1 x 10^20^ cm^-3^ | 1 x 10^20^ cm^-3^ |
| Effective density of states for valence band | 1 x 10^20^ cm^-3^ | 1 x 10^20^ cm^-3^ |
| Electron mobility | 2 cm^2^/(V·s) | 0.01 cm^2^/(V·s) |
| Hole mobility | 0.01 cm^2^/(V·s) | 0.1 cm^2^/(V·s) |
| Thickness | 50 nm | 50 nm |

## Table S10 Efficient four-terminal perovskite/Si tandem solar cells reported in the past three years

| **Year** | **Si type** | **PCE_Bottom_ (%)** | ***E_g,TOP_* (eV)** | **PCE_TOP_ (%)** | **PCE (%)** | **Refs** |
| --- | --- | --- | --- | --- | --- | --- |
| 2023 | PERL | 7.70 | 1.55 | 22.9 | 30.3 | [S38] |
| 2024 | HIT BC | 12.71 | 1.67 | 20.39 | 33.10 | [S36] |
| 2024 | HIT | 9.09 | 1.68 | 20.29 | 29.38 | [S37] |
| 2025 | HIT | 11.30 | 1.67 | 19.80 | 31.10 | [S32] |
| 2025 | HIT | 10.81 | 1.68 | 21.33 | 32.14 | [S33] |
| 2025 | TOPCON | 11.83 | 1.70 | 17.57 | 29.40 | [S34] |
| 2025 | TOPCON | 8.8 | 1.68 | 20.4 | 29.20 | [S35] |
| **2025** | **TOPCON** | **9.25** | **1.67** | **21.95** | **30.91** | **This work** |

# Supplementary References

1. A. Richter, J. Benick, M. Hermle, Boron emitter passivation with Al_\bf 2_O _\bf 3_ and Al_\bf 2_O_\bf 3_/SiN_\bm x_ stacks using ALD Al_\bf 2_O _\bf 3_. IEEE J. Photovoltaics **3**(1), 236–245 (2013). <https://doi.org/10.1109/jphotov.2012.2226145>
2. F. Feldmann, M. Bivour, C. Reichel, M. Hermle, S.W. Glunz, Passivated rear contacts for high-efficiency n-type Si solar cells providing high interface passivation quality and excellent transport characteristics. Sol. Energy Mater. Sol. Cells **120**, 270–274 (2014). <https://doi.org/10.1016/j.solmat.2013.09.017>
3. F. Feldmann, M. Simon, M. Bivour, C. Reichel, M. Hermle et al., Efficient carrier-selective p- and n-contacts for Si solar cells. Sol. Energy Mater. Sol. Cells **131**, 100–104 (2014). <https://doi.org/10.1016/j.solmat.2014.05.039>
4. H. Hu, S. X. An, Y. Li, S. Orooji, R. Singh et al., Triple-junction perovskite–perovskite–silicon solar cells with power conversion efficiency of 24.4%. Energy Environ. Sci. **17**(8), 2800-2814 (2024). <https://doi.org/10.1039/D3EE03687A>
5. C. Liu, Y. Yang, H. Chen, J. Xu, A. Liu et al., Bimolecularly passivated interface enables efficient and stable inverted perovskite solar cells. Science **382**(6672), 810–815 (2023). <https://doi.org/10.1126/science.adk1633>
6. Y. Wu, P. Zheng, J. Peng, M. Xu, Y. Chen et al., 27.6% perovskite/c-Si tandem solar cells using industrial fabricated TOPCon device. Adv. Energy Mater. **12**(27), 2200821 (2022). <https://doi.org/10.1002/aenm.202200821>
7. G. Bartholazzi Lugao De Carvalho, Hole-selective passivating contacts: From process development to application (2025).
8. K. Wei, L. Yang, J. Deng, Z. Luo, X. Zhang et al., Facile exfoliation of the perovskite thin film for visualizing the buried interfaces in perovskite solar cells. ACS Appl. Energy Mater. **5**(6), 7458–7465 (2022). <https://doi.org/10.1021/acsaem.2c00948>
9. J. Peng, D. Walter, Y. Ren, M. Tebyetekerwa, Y. Wu et al., Nanoscale localized contacts for high fill factors in polymer-passivated perovskite solar cells. Science **371**(6527), 390–395 (2021). <https://doi.org/10.1126/science.abb8687>
10. H. Zhan, V. Ahmad, A. Mayon, G. Dansoa Tabi, A.D. Bui et al., Physics-based extraction of material parameters from perovskite experiments *via* Bayesian optimization. Energy Environ. Sci. **17**(13), 4735–4745 (2024). <https://doi.org/10.1039/d4ee00911h>
11. S. Rühle, Tabulated values of the Shockley–Queisser limit for single junction solar cells. Sol. Energy **130**, 139–147 (2016). <https://doi.org/10.1016/j.solener.2016.02.015>
12. S. Wang, P. Wang, B. Shi, C. Sun, H. Sun et al., Inorganic perovskite surface reconfiguration for stable inverted solar cells with 20.38% efficiency and its application in tandem devices. Adv. Mater. **35**(28), e2300581 (2023). <https://doi.org/10.1002/adma.202300581>
13. N. Ren, P. Wang, J. Jiang, R. Li, W. Han et al., Multifunctional additive CdAc(2) for efficient perovskite-based solar cells. Adv. Mater. **35**(32), e2211806 (2023). <https://doi.org/10.1002/adma.202211806>
14. S. Zhang, F. Ye, X. Wang, R. Chen, H. Zhang et al., Minimizing buried interfacial defects for efficient inverted perovskite solar cells. Science **380**(6643), 404–409 (2023). <https://doi.org/10.1126/science.adg3755>
15. Z. Li, X. Sun, X. Zheng, B. Li, D. Gao et al., Stabilized hole-selective layer for high-performance inverted p-i-n perovskite solar cells. Science **382**(6668), 284–289 (2023). <https://doi.org/10.1126/science.ade9637>
16. S.S. Mali, J.V. Patil, J.-Y. Shao, Y.-W. Zhong, S.R. Rondiya et al., Phase-heterojunction all-inorganic perovskite solar cells surpassing 21.5% efficiency. Nat. Energy **8**(9), 989–1001 (2023). <https://doi.org/10.1038/s41560-023-01310-y>
17. Y. Zhao, C. Wang, T. Ma, L. Zhou, Z. Wu et al., Reduced 0.418 V *V*_OC_-deficit of 1.73 eV wide-bandgap perovskite solar cells assisted by dual chlorides for efficient all-perovskite tandems. Energy Environ. Sci. **16**(5), 2080–2089 (2023). <https://doi.org/10.1039/d2ee04087e>
18. X. Liu, J. Zhang, L. Tang, J. Gong, W. Li et al., Over 28% efficiency perovskite/Cu(*InGa*)Se_2_ tandem solar cells: highly efficient sub-cells and their bandgap matching. Energy Environ. Sci. **16**(11), 5029–5042 (2023). <https://doi.org/10.1039/D3EE00869J>
19. X. Zheng, Z. Li, Y. Zhang, M. Chen, T. Liu et al., Co-deposition of hole-selective contact and absorber for improving the processability of perovskite solar cells. Nat. Energy **8**(5), 462–472 (2023). <https://doi.org/10.1038/s41560-023-01227-6>
20. S. Mariotti, E. Köhnen, F. Scheler, K. Sveinbjörnsson, L. Zimmermann et al., Interface engineering for high-performance, triple-halide perovskite-silicon tandem solar cells. Science **381**(6653), 63–69 (2023). <https://doi.org/10.1126/science.adf5872>
21. G. Yang, Z.J. Yu, M. Wang, Z. Shi, Z. Ni et al., Shunt mitigation toward efficient large-area perovskite-silicon tandem solar cells. Cell Rep. Phys. Sci. **4**(10), 101628 (2023). <https://doi.org/10.1016/j.xcrp.2023.101628>
22. Z. Xiong, L. Wu, X. Zhou, S. Yang, Z. Liu et al., Constructing tin oxides interfacial layer with gradient compositions for efficient perovskite/silicon tandem solar cells with efficiency exceeding 28%. Small **20**(15), 2308024 (2024). <https://doi.org/10.1002/smll.202308024>
23. Z. Liu, H. Li, Z. Chu, R. Xia, J. Wen et al., Reducing perovskite/C60 interface losses *via* sequential interface engineering for efficient perovskite/silicon tandem solar cell. Adv. Mater. **36**(8), 2308370 (2024). <https://doi.org/10.1002/adma.202308370>
24. H. Luo, X. Zheng, W. Kong, Z. Liu, H. Li et al., Inorganic framework composition engineering for scalable fabrication of perovskite/silicon tandem solar cells. ACS Energy Lett. **8**(12), 4993–5002 (2023). <https://doi.org/10.1021/acsenergylett.3c02002>
25. Q. Xu, B. Shi, Y. Li, J. Liu, Y. Li et al., Diffusible capping layer enabled homogeneous crystallization and component distribution of hybrid sequential deposited perovskite. Adv. Mater. **36**(5), e2308692 (2024). <https://doi.org/10.1002/adma.202308692>
26. G. Wang, J. Zheng, W. Duan, J. Yang, M.A. Mahmud et al., Molecular engineering of hole-selective layer for high band gap perovskites for highly efficient and stable perovskite-silicon tandem solar cells. Joule **7**(11), 2583–2594 (2023). <https://doi.org/10.1016/j.joule.2023.09.007>
27. L. Qiao, T. Ye, T. Wang, W. Kong, R. Sun et al., Freezing halide segregation under intense light for photostable perovskite/silicon tandem solar cells. Adv. Energy Mater. **14**(7), 2302983 (2024). <https://doi.org/10.1002/aenm.202302983>
28. A.S. Subbiah, L.V. Torres Merino, A.R. Pininti, V. Hnapovskyi, S. Mannar et al., Enhancing the performance of blade-coated perovskite/silicon tandems *via* molecular doping and interfacial energy alignment. ACS Energy Lett. **9**(2), 727–731 (2024). <https://doi.org/10.1021/acsenergylett.4c00070>
29. S. Li, Z. Zheng, J. Ju, S. Cheng, F. Chen et al., A generic strategy to stabilize wide bandgap perovskites for efficient tandem solar cells. Adv. Mater. **36**(9), 2307701 (2024). <https://doi.org/10.1002/adma.202307701>
30. Z. Fang, B. Deng, Y. Jin, L. Yang, L. Chen et al., Surface reconstruction of wide-bandgap perovskites enables efficient perovskite/silicon tandem solar cells. Nat. Commun. **15**(1), 10554 (2024). <https://doi.org/10.1038/s41467-024-54925-4>
31. Z. Wang, Z. Han, X. Chu, H. Zhou, S. Yu et al., Regulation of wide bandgap perovskite by rubidium thiocyanate for efficient silicon/perovskite tandem solar cells. Adv. Mater. **36**(50), e2407681 (2024). <https://doi.org/10.1002/adma.202407681>
32. M. Hu, S. Du, Z. Yu, G. Chen, J. Liang et al., Improving efficiency and stability of wide-bandgap perovskite solar cells and four-terminal tandems with *Iso*-propylammonium 2D passivator. Chem. Eng. J. **505**, 159453 (2025). <https://doi.org/10.1016/j.cej.2025.159453>
33. H. Li, B. Gao, L. Yang, S. Wang, K. Gao et al., Magnesium oxide buffer layer for over 32% efficiency four-terminal perovskite/silicon tandem solar cells. Adv. Energy Mater. 2501762 (2025). <https://doi.org/10.1002/aenm.202501762>
34. W. Chai, W. Zhu, H. Xi, D. Chen, H. Dong et al., Buried interface regulation with TbCl(3) for highly-efficient all-inorganic perovskite/silicon tandem solar cells. Nano-Micro Lett. **17**(1), 244 (2025). <https://doi.org/10.1007/s40820-025-01763-8>
35. X. Guo, Z. Ying, X. Li, M. Zhang, S. Su et al., Oblique-angle damage-free evaporation of silicon oxide electron-selective passivation contacts for efficient and stable perovskite and perovskite/TOPCon tandem solar cells. Adv. Energy Mater. **15**(4), 2403021 (2025). <https://doi.org/10.1002/aenm.202403021>
36. Z. Fang, B. Deng, Y. Jin, L. Yang, L. Chen et al., Surface reconstruction of wide-bandgap perovskites enables efficient perovskite/silicon tandem solar cells. Nat. Commun. **15**(1), 10554 (2024). <https://doi.org/10.1038/s41467-024-54925-4>
37. C. Li, S. Dogan, Y. Li, H. Zhang, S. Tang et al., Mitigating VOC loss in single-junction and four-terminal tandem perovskite/Si photovoltaics with D-a phthalocyanines layers. Adv. Energy Mater. **15**(4), 2402856 (2025). <https://doi.org/10.1002/aenm.202402856>
38. T. Duong, T. Nguyen, K. Huang, H. Pham, S.G. Adhikari et al., Bulk incorporation with 4-methylphenethylammonium chloride for efficient and stable methylammonium-free perovskite and perovskite-silicon tandem solar cells. Adv. Energy Mater. **13**(9), 2203607 (2023). <https://doi.org/10.1002/aenm.202203607>
